# Homology and osteological correlates of pedal muscles among extant sauropsids

**DOI:** 10.1111/joa.13307

**Published:** 2020-09-24

**Authors:** Soki Hattori, Takanobu Tsuihiji

**Affiliations:** ^1^ Institute of Dinosaur Research Fukui Prefectural University Eiheiji‐cho Fukui Japan; ^2^ Fukui Prefectural Dinosaur Museum Katsuyama Fukui Japan; ^3^ Department of Geology and Paleontology National Museum of Nature and Science Tsukuba Ibaraki Japan; ^4^ Department of Earth and Planetary Science The University of Tokyo Bunkyo‐ku Tokyo Japan

**Keywords:** Archosauria, Aves, Crocodilia, homology, Lepidosauria, muscle, osteological correlates, pes, Sauropsida, Testudines

## Abstract

Archosaurs displayed an evolutionary trend toward increasing bipedalism in their evolutionary history, that is, forelimbs tend to be reduced in contrast to the development of hindlimbs becoming major weight‐bearing and locomotor appendages. The archosaurian locomotion has been extensively discussed based on their limb morphology because the latter reflects their locomotor modes very well. However, despite some attempts of reconstructing the hindlimb musculature in Archosauria, that of the most distal portion, the pes, has often been neglected. In order to rectify this trend, detailed homologies of pedal muscles among sauropsids were established based on dissections and literature reviews of adult conditions. As a result, homologies of some pedal muscles between non‐avian sauropsids and avians were revised, challenging classical hypotheses. The present new hypothesis postulates that the avian m. tibialis cranialis and non‐avian m. extensor digitorum longus, as well as the avian m. extensor digitorum longus and non‐avian m. tibialis anterior, are homologous with each other, respectively. This is more plausible because it requires no drastical change in the attachment sites between the avian and non‐avian homologues unlike the classical hypothesis. Many interosseous muscles in non‐archosaurian sauropsids that have long been regarded as a part of short digital extensors or flexors are also divided into multiple distinct muscles so that they can be homologized with short pedal muscles among all sauropsids. In addition, osteological correlates of attachments are identified for most of the pedal muscles, contributing to future attempts of reconstruction of this muscle system in fossil archosaurs.

## Graphical abstract

Despite some attempts of reconstructing the hindlimb musculature in Archosauria, that of the most distal portion, the pes, has often been neglected. In order to rectify this trend, detailed homologies of pedal muscles among sauropsids were established based on dissections and literature reviews of adult conditions. As a result, homologies of some pedal muscles between non‐avian sauropsids and avians were revised, challenging classical hypotheses. In addition, osteological correlates of attachments are identified for most of the pedal muscles, contributing to future attempts of reconstruction of this muscle system in fossil archosaurs.

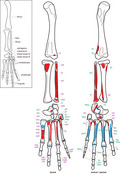

## 1 INTRODUCTION

Since Romer’s (1923a, 1923b, 1927) pioneering work on the archosaurian limb myology based on comprehensive examinations on the relationship between osteological features and limb postures in lepidosaurs, crocodilians, and dinosaurs, locomotion in Archosauria has been extensively discussed in paleontology. Moreover, the recognition of avians (birds) as the descendants of Mesozoic theropod dinosaurs has led us to further understanding of major evolutionary changes in the limb morphology and function during the entire evolutionary history of Archosauria (Gatesy and Dial, 1996; Farlow *et al*., 2000; Hutchinson, 2009; Gauthier *et al*., 2011). Among archosaurs, the hindlimb of theropod dinosaurs is specialized for bipedal locomotion, which is inherited by extant avians. Although the main role of the hindlimb has not been altered from terrestrial locomotion within Theropoda, detailed morphology of this body part has been extensively modified on the line to Aves (Gatesy, 2002).

The musculature is fundamental for understanding locomotor functions and, thus, is the most often reconstructed aspect of the soft tissue anatomy in fossil vertebrates (e.g. Romer, 1927; Carrano and Hutchinson, 2002; Persons and Currie, 2011). Some muscle attachments leave distinct morphological signatures on bone surfaces, namely, osteological correlates, that are often preserved on fossils, such as muscle scars, crests, tubercles, trochanters, and smooth surfaces representing attachments or courses of muscles and/or tendons (Hutchinson, 2002; Burch, 2014).

Mostly based on the osteological correlates described above, reconstructions of the musculature in extinct vertebrates have been attempted for over 130 years. Relatively recently, a phylogenetically rigorous methodology for reconstructing soft anatomy in fossils was proposed (Bryant and Russell, 1993; Witmer, 1995). The methodology, widely known as the extant phylogenetic bracketing approach, is firmly based on homologous structures between at least two extant outgroups of the fossil taxon of interest and provides a criterion for rigorously establishing the limits of inferences (Witmer, 1995).

Despite some previous attempts of reconstructing the hindlimb musculature in archosaurs, the most distal portion of this body part, the pes, has often been neglected. Among many muscles associated with the pes, only those arising from more proximal portion of the hindlimb, i.e., thigh and crus, have been reconstructed, with intrinsic pedal muscles arising from and inserting on the pedal bones only briefly mentioned in past studies (Dilkes, 2000; Carrano and Hutchinson, 2002; Hutchinson, 2002). This trend is mainly because of the greater complexity of the skeleton and musculature in the pes compared to the more proximal portion, in which both bones and muscles are large and can be dissected more easily. In addition, Romer’s (1923a, 1923b) homology hypotheses of hindlimb muscles, which have been the basis for muscle reconstructions in fossil archosaurs even in recent years, did not deal with the distal segment of the hindlimb.

In order to infer evolutionary changes in the pedal muscles in Archosauria, their detailed homologies among sauropsid taxa need to be established. The first comprehensive review of the pedal musculature among extant non‐avian sauropsids (or commonly called ‘reptiles’) was the monograph of Gadow (1882). He established major divisions of pedal muscles, naming them with Roman numerals with further subdivisions indicated with Greek letters. In Gadow (1882), attachment areas of these muscles on bones were briefly described but were not figured, whereas the details of the innervation pattern of the pedal muscles were figured for *Alligator mississippiensis* only. More comprehensive descriptions of the innervation pattern of the pedal musculature were provided in the monographs by Ribbing (1909, 1938). However, he recognized only major divisions of muscles unlike Gadow (1882), focusing on homologies of such divisions among sauropsids and amphibians. Later, more detailed myological descriptions of the pes were undertaken for lepidosaurs (Russell and Bauer, 2008), testudines (Walker, 1973), crocodilians (Cong *et al*., 1998; Suzuki *et al*., 2011), and avians (Fujioka, 1962; Vanden Berge, 1975). Although these more recent descriptions provide detailed information on the hindlimb musculature including the pedal region, each study was focused on a narrow taxonomic range, with little consideration for homologies of muscles among taxa, especially between non‐avians and avians.

In addition, osteological correlates of muscle attachments have not been described in most anatomical studies. In the past, osteological correlates were examined in detail in studies specifically intended for paleontological applications (e.g. Hutchinson, 2001a, 2001b). However, as mentioned above, such a study is still lacking on the pedal region. For these reasons, the aim of the present study is to describe muscles associated with pedes in detail, compare their morphology, establish their homologies among extant sauropsids, and describe their osteological correlates for future paleontological applications.

## 2 MATERIALS AND METHODS

**Figure 1:**
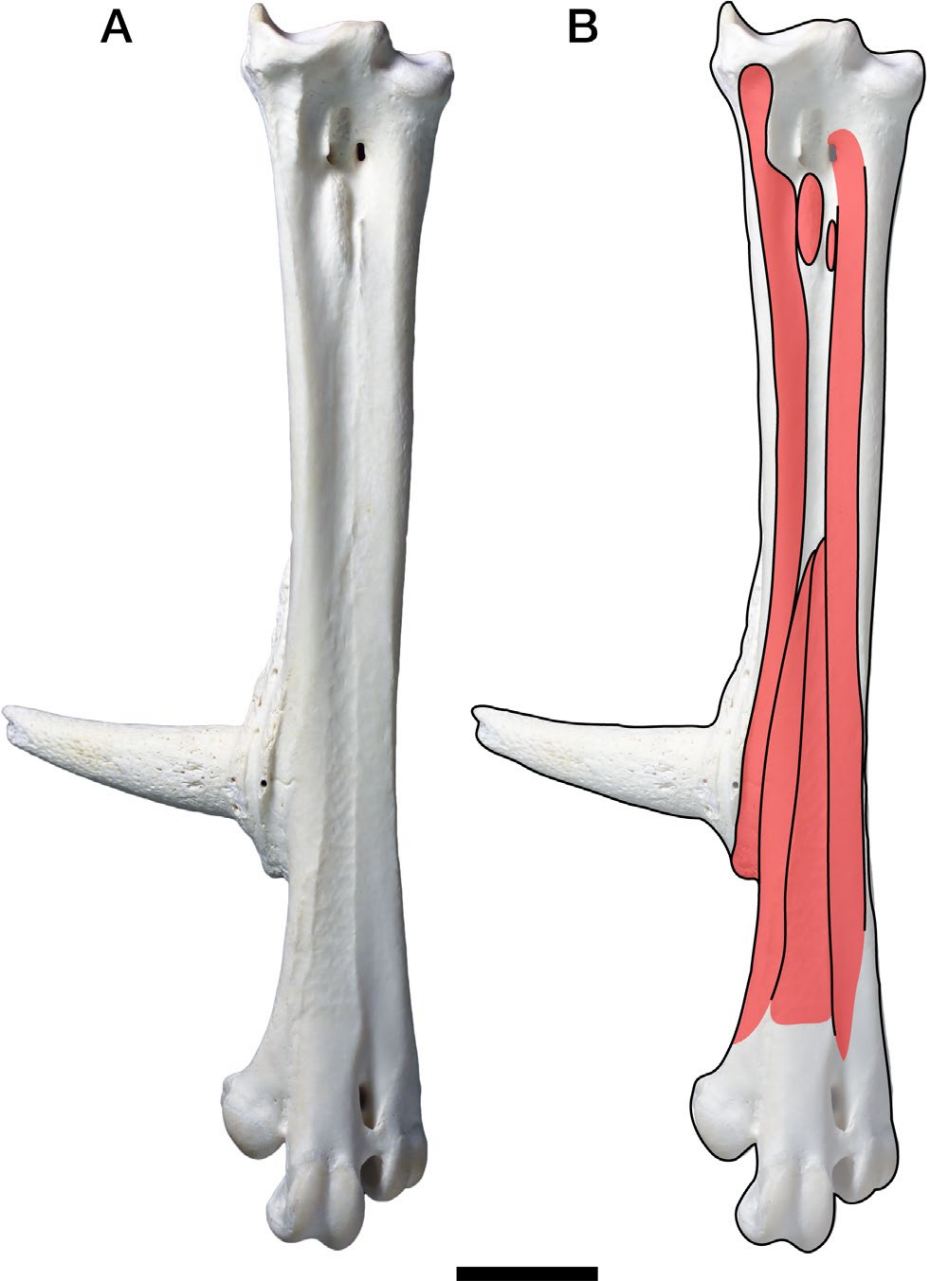
Osteological correlates on the tarsometatarsus of *Gallus gallus*. (A) Left tarsometatarsus in dorsal view. (B) Drawing of muscle attachment sites (red) and their osteological correlates (black)

To observe the morphology of the pedal muscles in detail, the following specimens were dissected (the number in each bracket indicates the number of dissected specimens for each species): squamates *Iguana iguana* [2] and *Varanus indicus* [1], turtle *Chelydra serpentina* [1], crocodilians *Paleosuchus palpebrosus* [1] and *Crocodylus porosus* [2], and avians *Gallus gallus* [1] and *Grus japonensis* [1]. These specimens were fixed and preserved in aqueous solution of 70% ethanol except for *Paleosuchus* and one of *Crocodylus*, which were dissected without fixation. Positions and morphology of osteological correlates for muscle attachments were also directly observed in each dissected specimen (Figure 1).

The nomenclature of the muscles follows Russell and Bauer (2008) for lepidosaurs, Walker (1973) for testudines, Suzuki *et al*. (2011) for crocodilians, and Vanden Berge and Zweers (1993) for avians. Putatively homologous muscles are described under each standardized name, which mostly follows nomenclature used in Suzuki *et al*. (2011). Although Suzuki *et al*. (2011) is the most detailed description of the crocodilian hindlimb muscles at this time, it is written in Japanese and thus difficult to understand for non‐Japanese researchers. Therefore, to help understanding, names of corresponding muscles used in other studies (Gadow, 1882; Ribbing, 1938; Cong *et al*., 1998) are indicated in Table 1. Similarly, corresponding muscle names in other studies are also indicated for lepidosaurs in Table 2, testudines in Table 3, and avians in Table 4, respectively.

**Table 1:** Synonymy of crocodilian hindlimb muscles among four previous studies

Standardized muscle name	Gadow (1882)	Ribbing (1938)	Cong *et al*. (1998)	Suzuki *et al*. (2011)
M. tibialis cranialis	M. extensor longus digitorum	M. extensor digitorum communis	M. extensor digitorum longus, long head, or lateral head	M. extensor digitorum longus
M. extensor digitorum longus	M. tibialis anticus	M. extensor tarsi tibialis	M. extensor digitorum longus, short head, or medial head	M. tibialis anterior
M. peroneus longus	M. peroneus posterior, in part	M. extensor tarsi fibularis, in part	M. peroneus longus	M. peroneus longus
M. peroneus brevis	M. peroneus anterior	M. extensor tarsi fibularis, in part	M. peroneus brevis	M. peroneus brevis
M. adductor hallucis dorsalis	M. extensor hallucis proprius	M. extensores breves, in part	M. extensor hallucis longus	M. adductor hallucis dorsalis
M. extensor digitorum brevis	Nr. II α	M. extensores breves, in part	M. extensor hallucis brevis, in part	M. extensor digitorum I, II et III
	Nr. II β		M. extensor digitorum brevis, in part (pars proximalis of digit II)	
	Nr. II γ		M. extensor digitorum brevis, in part (pars proximalis of digit III)	
M. extensor hallucis brevis	Nr. IV α	M. extensores breves, in part	M. extensor hallucis brevis, in part	M. extensor hallucis brevis
M. extensor digiti II	Nr. IV β	M. extensores breves, in part	M. extensor digitorum brevis, in part	M. extensor digiti II
M. extensor digiti III	Nr. IV γ	M. extensores breves, in part	M. extensor digitorum brevis, in part	M. extensor digiti III
M. extensor digiti IV	Nr. III	M. extensores breves, in part	M. extensor digitorum brevis, in part	M. extensor digiti IV
M. interosseous dorsalis digiti II	Nr. V α	M. extensores breves, in part	Mm. interossei dorsales, in part (first slip)	M. interosseous dorsalis digiti II
M. interosseous dorsalis digiti III	Nr. V β	M. extensores breves, in part	Mm. interossei dorsales, in part (second slip)	M. interosseous dorsalis digiti III
M. interosseous dorsalis digiti IV	Nr. V γ	M. extensores breves, in part	–	M. interosseous dorsalis digiti IV
M. gastrocnemius	M. gastrocnemius, in part	M. gastrocnemius	M. gastrocnemius	M. gastrocnemius
M. flexor hallucis longus	M. gastrocnemius, in part	M. flexor profundus, in part	M. flexor hallucis longus	M. flexor digitorum longus
M. flexor digitorum longus	M. flexor longus digitorum	M. flexor profundus, in part	M. flexor digitorum longus	M. flexor hallucis longus
M. pronator profundus	M. tibialis‐posticus	M. pronator profundus	M. tibialis posterior	M. pronator profundus
	M. interosseous cruris		M. flexor digiti quarti brevis	
M. fibulocalcaneus	M. peroneus posterior, in part	M. flexor metatarsi V	M. pronator profundus	M. fibulocalcaneus
M. flexor digitorum brevis superficialis	Nr. VI	Mm. flexores breves superficiales	M. flexor digitorum brevis	M. flexor digitorum brevis superficialis
M. flexor digitorum brevis profundus	Nr. VII	M. flexor accesorius medialis	M. quadratus plantae	M. flexor digitorum brevis profundus
Mm. lumbricales	Nr. VIII, in part	M. flexor accesorius lateralis	Mm. lumbricales pedis, in part (superficial layer)	Mm. lumbricales
M. lumbricalis profundus	Nr. VIII, in part	Mm. contrahentes digitorum	Mm. lumbricales pedis, in part (deep layer)	M. lumbricalis profundus
M. flexor hallucis brevis	Nr. X β	Mm. flexores breves profundi, in part	M. flexor hallucis	M. flexor hallucis brevis superficialis
	Nr. X α		M. abductor hallucis	M. flexor hallucis brevis profundus
M. adductor digiti V	–	–	–	–
Mm. contrahentes	Nr. X γ	Mm. flexores breves profundi, in part	Mm. interossei plantares, in part (first slip)	M. flexor digiti II
	Nr. X δ		Mm. interossei dorsales, in part (pars plantares)	M. flexor digiti III
	Nr. X ε		Mm. interossei plantares, in part (second slip)	M. flexor digiti IV
M. adductor hallucis plantaris	Nr. XI α	Mm. interdigitales, in part	M. adductor hallucis	M. adductor hallucis plantaris
M. interosseous plantaris digiti II	Nr. XI β	Mm. interdigitales, in part	Mm. interossei plantares, in part (first slip)	M. interosseous plantaris digiti II
M. interosseous plantaris digiti III	Nr. XI γ	Mm. interdigitales, in part	Mm. interossei plantares, in part (third slip)	M. interosseous plantaris digiti III
M. abductor digiti IV	Nr. XII	Mm. interdigitales, in part	M. abductor digiti quarti	M. abductor digiti IV dorsalis
				M. abductor digiti IV plantaris

**Table 2:** Synonymy of lepidosaurian hindlimb muscles among three previous studies

Standardized muscle name	Gadow (1882)	Ribbing (1938)	Russell and Bauer (2008)
M. tibialis cranialis	M. extensor longus digitorum	M. extensor digitorum communis	M. extensor digitorum longus
M. extensor digitorum longus	M. tibialis anticus	M. extensor tarsi tibialis	M. tibialis anterior
M. peroneus longus	M. peroneus posterior	M. extensor tarsi fibularis, in part	M. peroneus longus
M. peroneus brevis	M. peroneus anterior	M. extensor tarsi fibularis, in part	M. peroneus brevis
M. adductor hallucis dorsalis	M. extensor hallucis proprius	Mm. extensores breves, in part	M. adductor et extensor hallucis et indicus
M. extensor digitorum brevis	Nr. II	Mm. extensores breves, in part	Mm. extensores digitores breves, in part
M. extensor hallucis brevis	Nr. IV α, in part	Mm. extensores breves, in part	Mm. extensores digitores breves, in part
M. extensor digiti II	Nr. IV α, in part	Mm. extensores breves, in part	Mm. extensores digitores breves, in part
M. extensor digiti III	Nr. IV α, in part	Mm. extensores breves, in part	Mm. extensores digitores breves, in part
M. extensor digiti IV	Nr. III	Mm. extensores breves, in part	Mm. extensores digitores breves, in part
M. interosseous dorsalis digiti II	Nr. IV α, in part	–	Mm. extensores digitores breves, in part
M. interosseous dorsalis digiti III	Nr. IV α, in part	–	Mm. extensores digitores breves, in part
M. interosseous dorsalis digiti IV	Nr. IV α, in part	–	Mm. extensores digitores breves, in part
M. gastrocnemius	M. gastrocnemius, caput tibiale	M. gastrocnemius, internus	M. femorotibial gastrocnemius
	M. gastrocnemius, caput femorale	M. gastrocnemius, externus	M. femoral gastrocnemius
M. flexor hallucis longus	M. flexor longus digitorum, in part	M. flexor accessorius medialis	M. flexor digitorum longus, in part
M. flexor digitorum longus	M. flexor longus digitorum, in part	M. flexor profundus	M. flexor digitorum longus, in part
		M. flexor digiti V	
M. pronator profundus	M. tibialis posticus	M. pronator profundus	M. pronator profundus
M. fibulocalcaneus	–	M. flexor metatarsi V	M. abductor digiti quinti
M. flexor digitorum brevis superficialis	Nr. VI	Mm. flexores breves superficiales	Mm. flexores digitores breves
			pfdl. calc.
M. flexor digitorum brevis profundus	Nr. VII, in part	M. flexor accessorius lateralis, in part	pfdl. met. 5
Mm. lumbricales	–	Mm. flexores digitorum breves profundi	Mm. lumbricales
M. lumbricalis profundus	–	M. flexor accessorius lateralis, in part	pfdl. ap.
M. flexor hallucis brevis	Nr. X α	Mm. contrahentes digitorum, in part	M. flexor hallucis
M. adductor digiti V	–	–	M. adductor digiti quinti
Mm. contrahentes	Nr. X β–ε	Mm. contrahentes digitorum, in part	Mm. contrahentes
M. adductor hallucis plantaris	Nr. XI α	Mm. interdigitales, in part	Mm. interossei plantares, in part
M. interosseous plantaris digiti II	Nr. XI β	Mm. interdigitales, in part	Mm. interossei dorsales, in part
			Mm. interossei plantares, in part
M. interosseous plantaris digiti III	Nr. XI γ	Mm. interdigitales, in part	Mm. interossei dorsales, in part
			Mm. interossei plantares, in part
M. abductor digiti IV	Nr. IV β	–	Mm. extensores digitores breves, in part

**Table 3:** Synonymy of testudine hindlimb muscles among three previous studies

Standardized muscle name	Gadow (1882)	Ribbing (1938)	Walker (1973)
M. tibialis cranialis	M. extensor longus digitorum	M. extensor digitorum communis	M. extensor digitorum communis
M. extensor digitorum longus	M. tibialis anticus	M. extensor tarsi tibialis	M. tibialis anterior
M. peroneus longus	M. peroneus anterior	M. extensor tarsi fibularis, in part	M. peroneus anterior
M. peroneus brevis	M. peroneus posterior	M. extensor tarsi fibularis, in part	M. peroneus posterior
M. adductor hallucis dorsalis	M. extensor hallucis proprius	Mm. extensores breves, in part	M. extensor hallucis proprius
M. extensor digitorum brevis	Nr. II	Mm. extensores breves, in part	Mm. extensores digitorum breves
	Nr. VI		
	Nr. XI, in part		
M. extensor hallucis brevis	Nr. XI, in part	Mm. extensores breves, in part	M. abductor hallucis
M. extensor digiti II	Nr. XI, in part	Mm. extensores breves, in part	Mm. interossei dorsales, in part
M. extensor digiti III	Nr. XI, in part	Mm. extensores breves, in part	Mm. interossei dorsales, in part
M. extensor digiti IV	Nr. XI, in part	Mm. extensores breves, in part	Mm. interossei dorsales, in part
M. interosseous dorsalis digiti II	Nr. XI, in part	Mm. extensores breves, in part	Mm. interossei dorsales, in part
M. interosseous dorsalis digiti III	Nr. XI, in part	Mm. extensores breves, in part	Mm. interossei dorsales, in part
M. interosseous dorsalis digiti IV	Nr. XI, in part	Mm. extensores breves, in part	Mm. interossei dorsales, in part
M. gastrocnemius	M. gastrocnemius	M. gastrocnemius	M. gastrocnemius
M. flexor hallucis longus	M. flexor longus digitorum, in part	M. flexor profundus, in part	M. flexor digitorum longus, in part
M. flexor digitorum longus	M. flexor longus digitorum, in part	M. flexor profundus, in part	M. flexor digitorum longus, in part
		M. flexor accessorius lateralis	
M. pronator profundus	M. tibialis posticus	M. pronator profundus, in part	M. pronator profundus
M. fibulocalcaneus	–	–	–
M. flexor digitorum brevis superficialis	–	–	Plantar aponeurosis
M. flexor digitorum brevis profundus	–	–	–
Mm. lumbricales	Nr. VII, in part	Mm. flexores breves superficiales, in part	M. flexor digitorum communis sublimis
	Nr. VIII, in part		
M. lumbricalis profundus	Nr. VII, in part	Mm. flexores breves superficiales, in part	Mm. lumbricales
	Nr. VIII, in part		
M. flexor hallucis brevis	Nr. XI, in part	Mm. flexores breves profundi, in part	Mm. interossei plantares, in part
M. adductor digiti V	–	–	–
Mm. contrahentes	Nr. XI, in part	Mm. flexores breves profundi, in part	Mm. interossei plantares, in part
		M. contrahentes digitorum	
M. adductor hallucis plantaris	Nr. XI, in part	Mm. interdigitales, in part	Mm. interossei plantares, in part
M. interosseous plantaris digiti II	Nr. XI, in part	Mm. interdigitales, in part	Mm. interossei plantares, in part
M. interosseous plantaris digiti III	Nr. XI, in part	Mm. interdigitales, in part	Mm. interossei plantares, in part
M. abductor digiti IV	Nr. XI, in part	Mm. interdigitales, in part	Mm. interossei plantares, in part

**Table 4:** Synonymy of avian hindlimb muscles among three previous studies

Standardized muscle name	Fujioka (1962)	Vanden Berge (1975)	Vanden Berge and Zweers (1993)
M. tibialis cranialis	M. tibialis anterior	M. tibialis cranialis	M. tibialis cranialis
M. extensor digitorum longus	M. extensor digitorum longus	M. extensor digitorum longus	M. extensor digitorum longus
M. peroneus longus	M. peroneus longus	M. fibularis (peroneus) longus	M. fibularis (peroneus) longus
M. peroneus brevis	M. peroneus brevis	M. fibularis (peroneus) brevis	M. fibularis (peroneus) brevis
M. adductor hallucis dorsalis	–	–	–
M. extensor digitorum brevis	–	–	–
M. extensor hallucis brevis	M. extensor hallucis longus	M. extensor hallucis longus	M. extensor hallucis longus
M. extensor digiti II	–	–	–
M. extensor digiti III	M. extensor digiti terti	M. extensor brevis digiti III	M. extensor brevis digiti III
M. extensor digiti IV	–	–	–
M. interosseous dorsalis digiti II	M. abductor digiti secundi	M. abductor (extensor) digiti II	M. abductor digiti II
M. interosseous dorsalis digiti III	–	–	–
M. interosseous dorsalis digiti IV	M. adductor digiti quarti	M. extensor brevis digiti IV	M. extensor brevis digiti IV
M. gastrocnemius	M. gastrocnemius	M. gastrocnemius	M. gastrocnemius
M. flexor hallucis longus	M. flexor hallucls longus	M. flexor hallucis longus	M. flexor hallucis longus
	M. flexor perforans et perforatus digiti secundi	M. flexor perforans et perforatus digiti II	M. flexor perforans et perforatus digiti II
	M. flexor perforans et perforatus digiti tertii	M. flexor perforans et perforatus digiti III	M. flexor perforans et perforatus digiti III
	M. flexor perforatus digiti secundi	M. flexor perforatus digiti II	M. flexor perforatus digiti II
	M. flexor perforatus digiti terti	M. flexor perforatus digiti III	M. flexor perforatus digiti III
	M. flexor perforatus digiti quarti	M. flexor perforatus digiti IV	M. flexor perforatus digiti IV
M. flexor digitorum longus	M. flexor perforans digitorum profundus	M. flexor digitorum longus	M. flexor digitorum longus
	M. tibialis posterior	M. plantaris	M. plantaris
M. pronator profundus	–	–	–
M. fibulocalcaneus	–	–	–
M. flexor digitorum brevis superficialis	–	–	–
M. flexor digitorum brevis profundus	–	–	–
Mm. lumbricales	–	–	–
M. lumbricalis profundus	–	M. lumbricalis	M. lumbricalis
M. flexor hallucis brevis	–	–	–
M. adductor digiti V	–	–	–
Mm. contrahentes	–	–	–
M. adductor hallucis plantaris	M. flexor hallucis brevis	M. flexor hallucis brevis	M. flexor hallucis brevis
M. interosseous plantaris digiti II	M. adductor digiti secundi	M. adductor digiti II	M. adductor digiti II
M. interosseous plantaris digiti III	–	–	–
M. abductor digiti IV	M. abductor digiti quarti	M. abductor digiti IV	M. abductor digiti IV

To assess the muscle homology, the innervation pattern has often been considered as the most significant criterion. It is because that the nerve‐muscle specificity has been assumed to be more conservative phylogenetically than are other criteria, namely, the morphology and positions of muscle attachment sites and their mechanical functions. However, Straus (1946) reviewed this concept of nerve‐muscle specificity and concluded that resemblances in the innervation pattern across taxa merely result from general similarity in the development pattern, rather than reflecting any inherent and immutable connection between the muscle and nerve. Actually, some topologically similar pedal muscles with similar mechanical functions have also been found to have different innervation patterns (Russell and Bauer, 2008). For example, the detailed innervation patterns are often inconsistent among studies on crocodilians. One muscle, m. tibialis cranialis, whose homology among crocodilians is broadly accepted, is innervated by both n. peroneus superficialis and n. peroneus profundus according to Gadow (1882) but only by n. peroneus profundus according to Cong *et al*. (1998) and Suzuki *et al*. (2011). As another example, the distal portions of both fibular nerves form a loop, which gives rise to the branch supplying m. interosseous dorsalis digiti III in crocodilians (Gadow, 1882; S. Hattori, pers. obs.). In such a loop, it is difficult to assess which nerve is dominant one contributing to the branch. In these cases, therefore, muscles with different innervation patterns are not necessarily non‐homologous (Cunningham, 1881; Gadow, 1882). Accordingly, the innervation patterns were not used as the exclusive, primary criterion for the muscle homology in the present study. Instead, the congruence among the innervation patterns, the morphology and positions of attachment sites of muscles and similarities of their mechanical functions was regarded as the strongest evidence for the homology.

## 3 RESULTS

**Figure 2:**
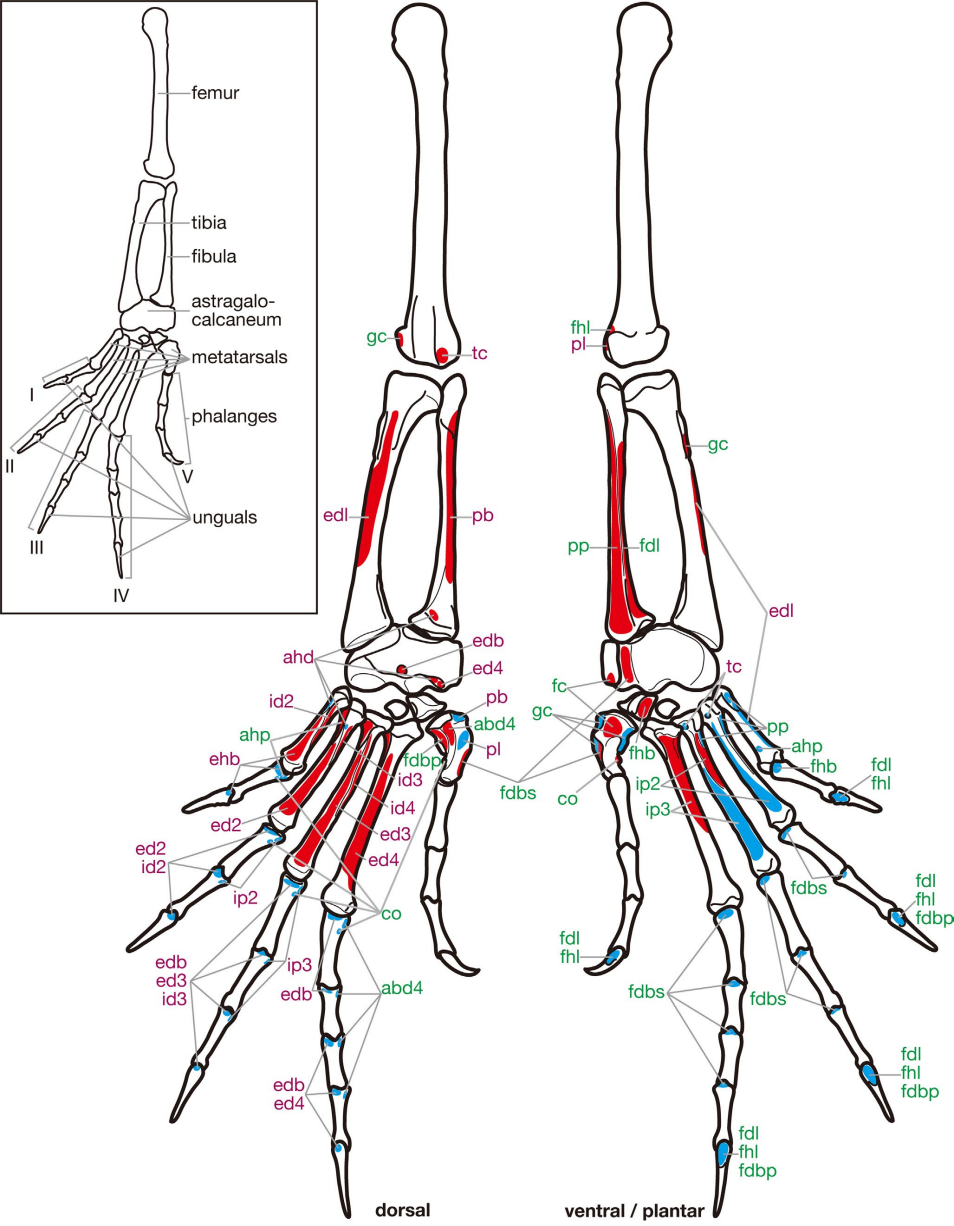
Hindlimb skeleton and attachment sites of pedal muscles in *Iguana iguana*. Muscle origins and insertions are indicated in red and blue, respectively. Names of dorsal and ventral/plantar muscles are indicated in purple and green, respectively. See Table 5 for abbreviations

**Figure 3:**
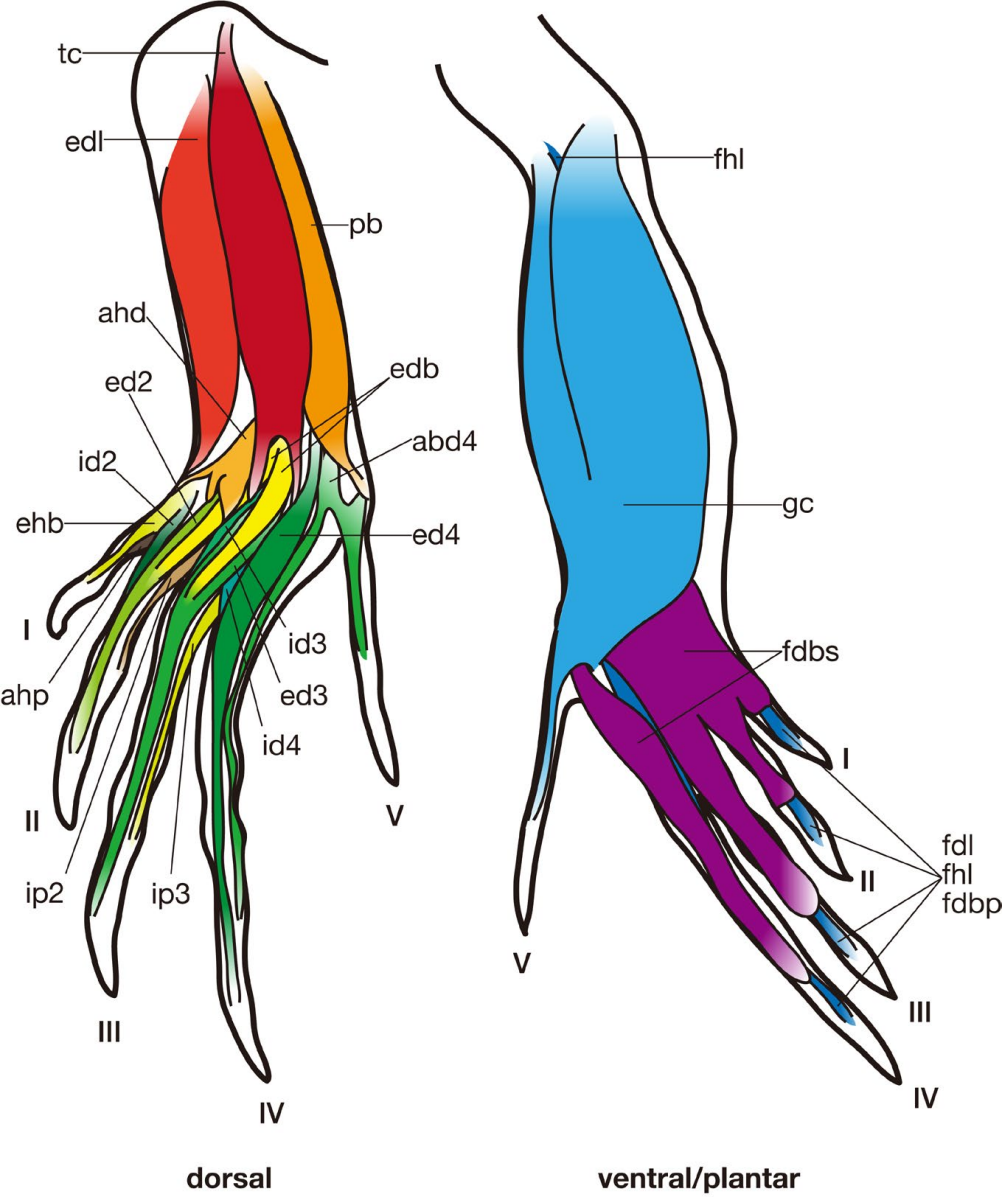
Semi‐schematic illustrations of the pedal muscles of *Iguana iguana* (modified after Russell and Bauer, 2008). Some muscles are omitted for simplicity. See Table 5 for abbreviations

**Figure 4:**
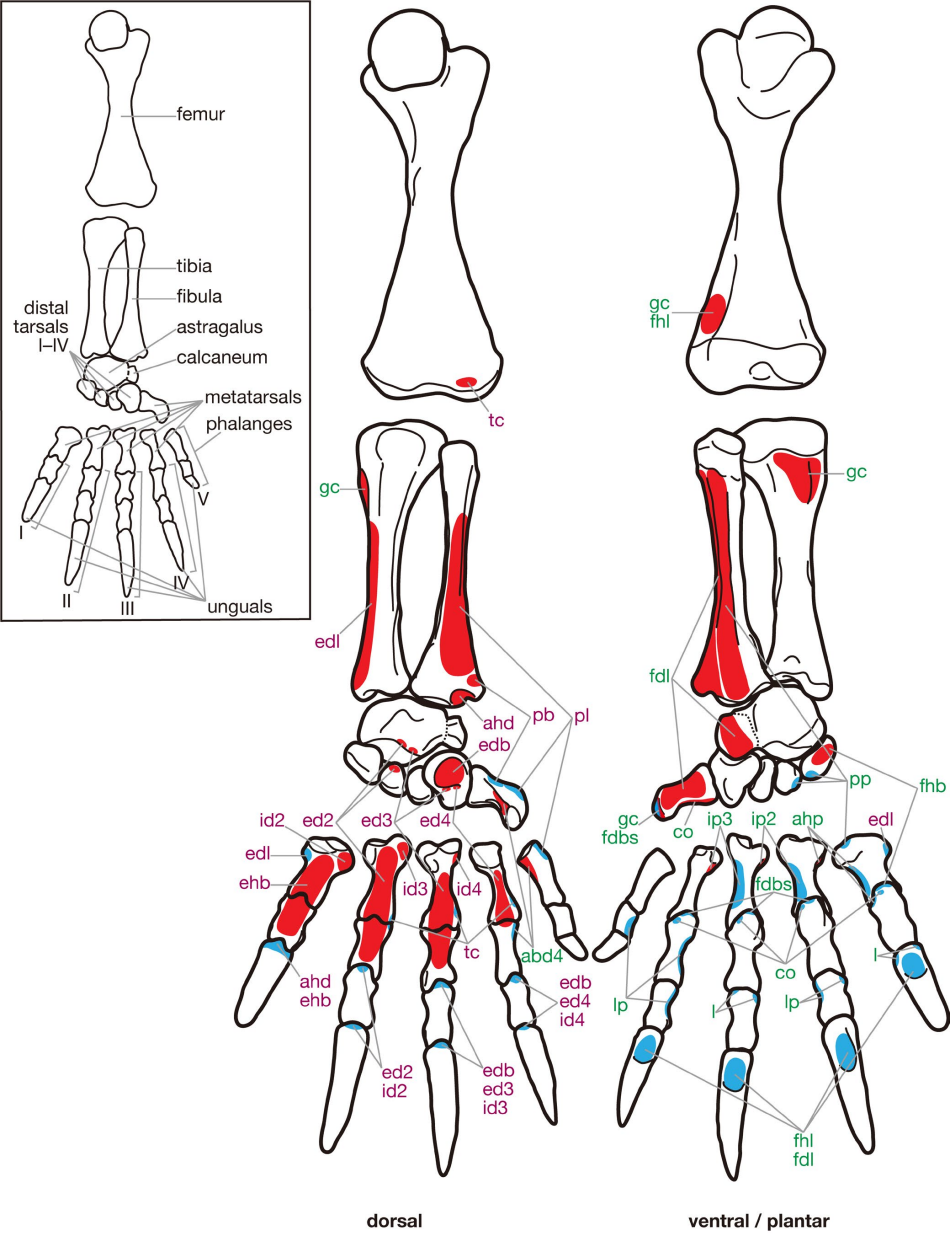
Hindlimb skeleton and attachment sites of pedal muscles in *Chelydra serpentina*. Muscle origins and insertions are indicated in red and blue, respectively. Names of dorsal and ventral/plantar muscles are indicated in purple and green, respectively. See Table 5 for abbreviations

**Figure 5:**
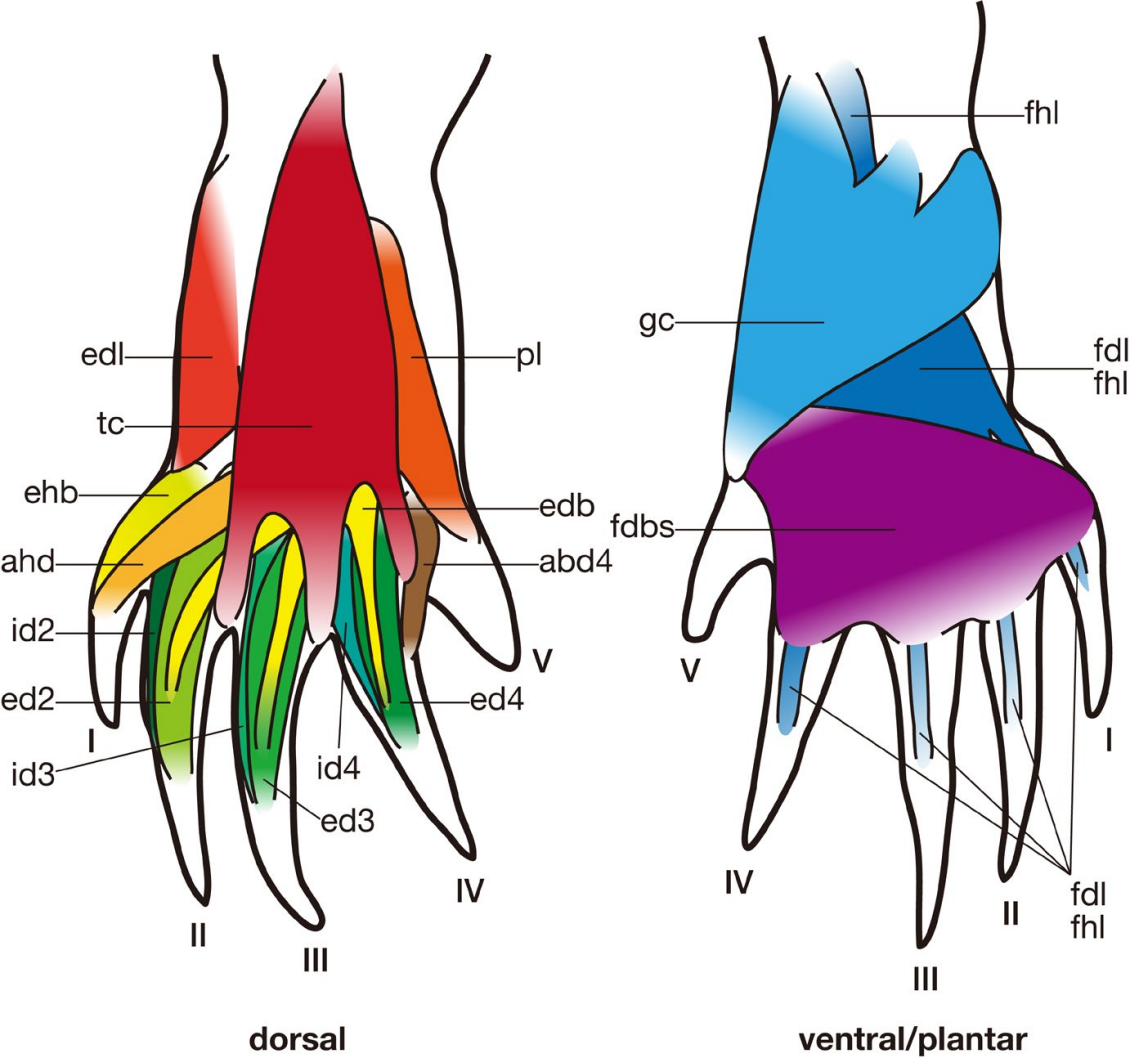
Semi‐schematic illustrations of the pedal muscles of *Chelydra serpentina* (modified after Walker, 1973). Some muscles are omitted for simplicity. See Table 5 for abbreviations

**Figure 6:**
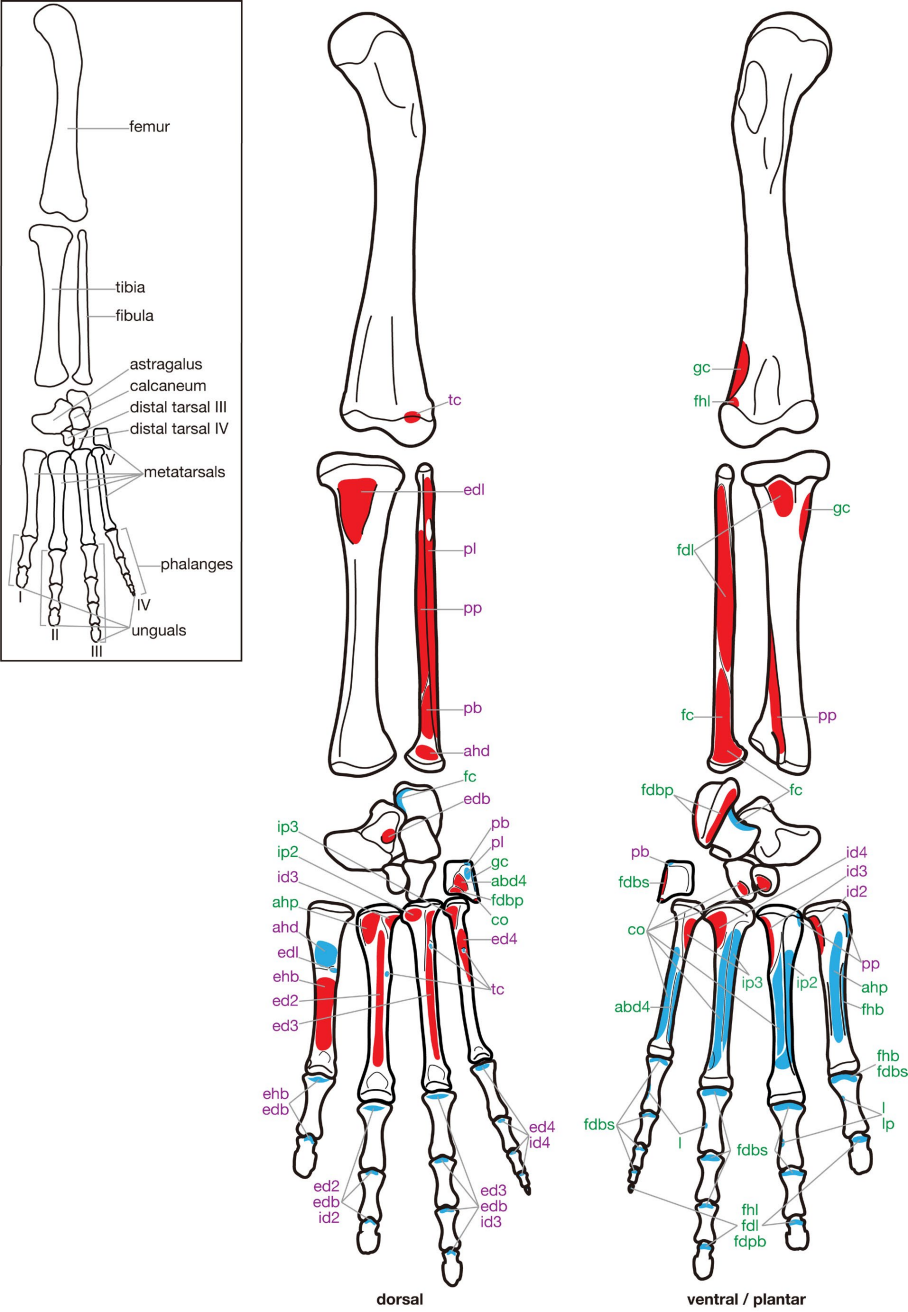
Hindlimb skeleton and attachment sites of pedal muscles in *Paleosuchus palpebrosus*. Muscle origins and insertions are indicated in red and blue, respectively. Names of dorsal and ventral/plantar muscles are indicated in purple and green, respectively. See Table 5 for abbreviations

**Figure 7:**
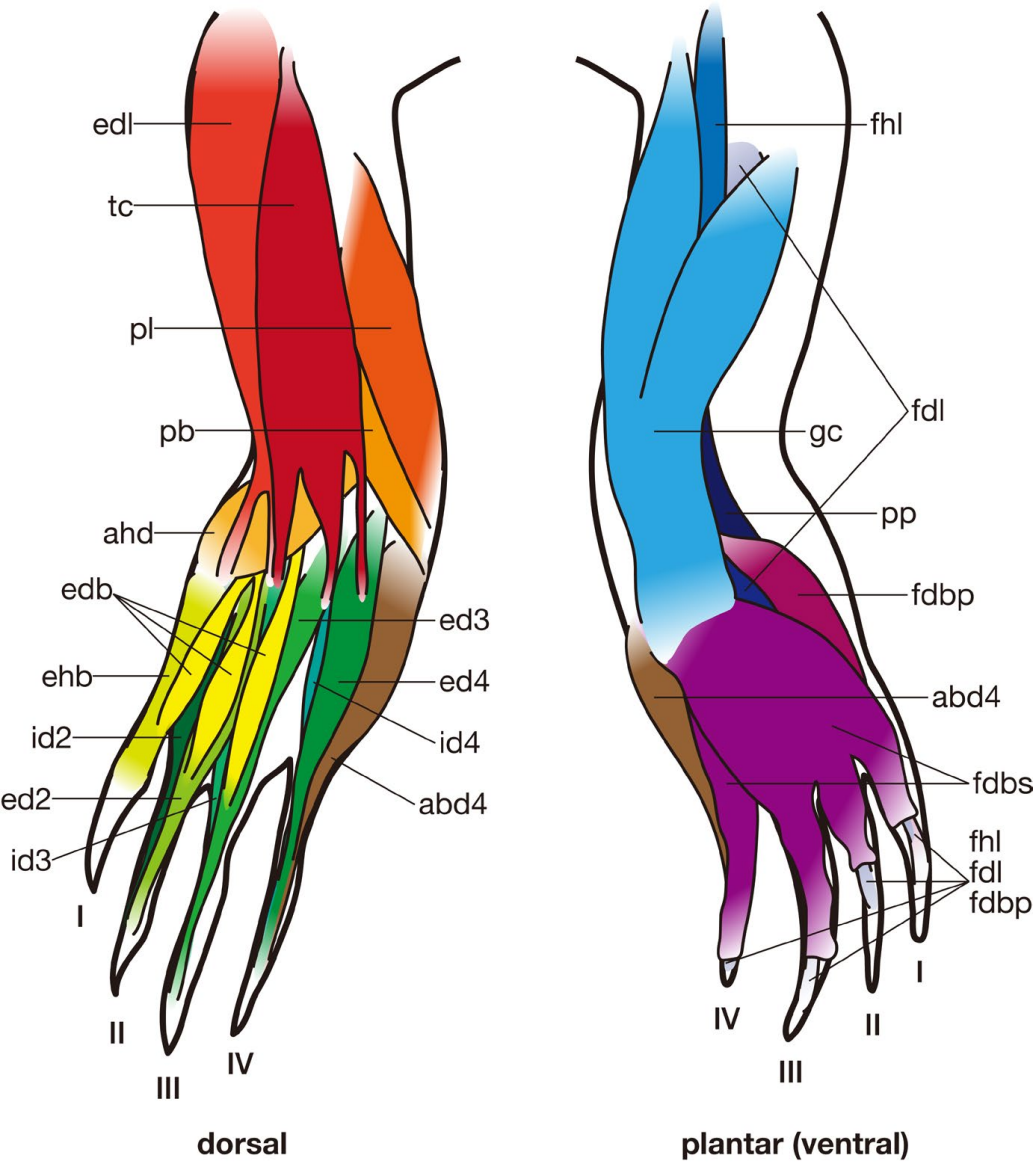
Pedal muscles of *Paleosuchus palpebrosus* (modified after Ribbing, 1938). Some muscles are omitted for simplicity. See Table 5 for abbreviations

**Figure 8:**
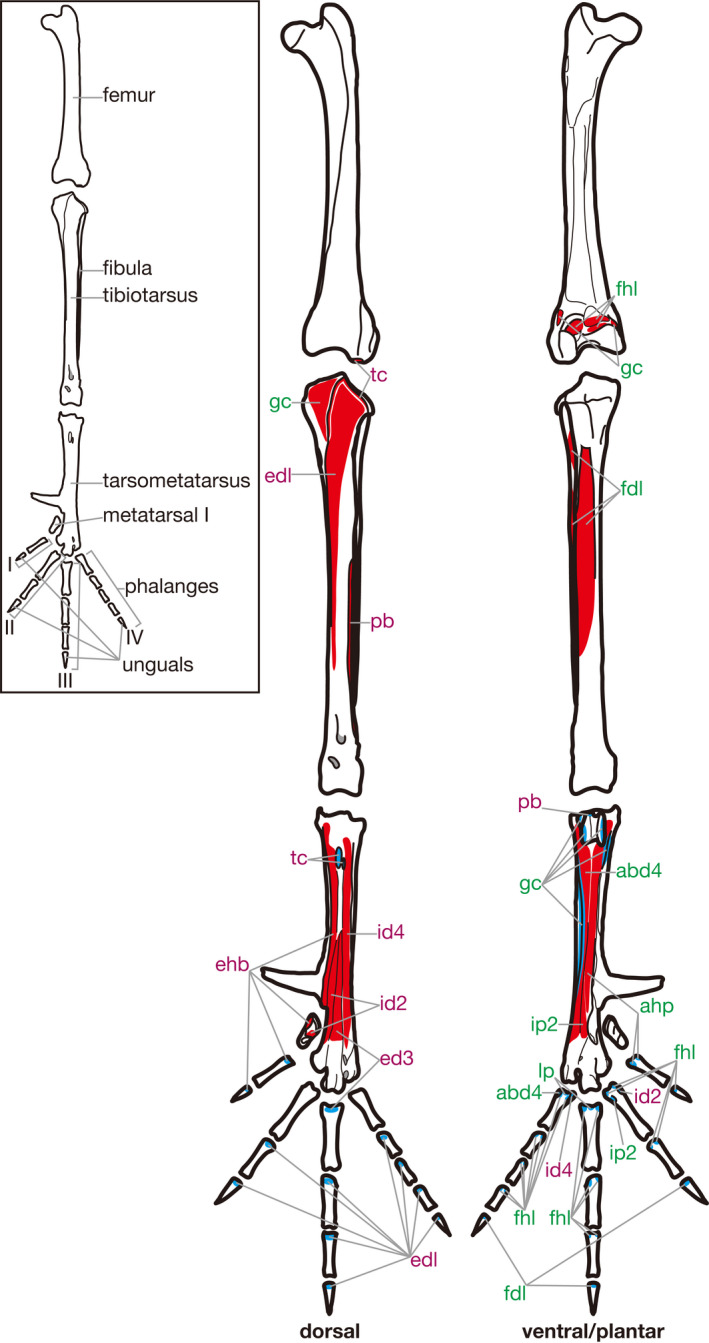
Hindlimb skeleton and attachment sites of pedal muscles in *Gallus gallus*. Muscle origins and insertions are indicated in red and blue, respectively. Names of dorsal and ventral/plantar muscles are indicated in purple and green, respectively. See Table 5 for abbreviations

**Figure 9:**
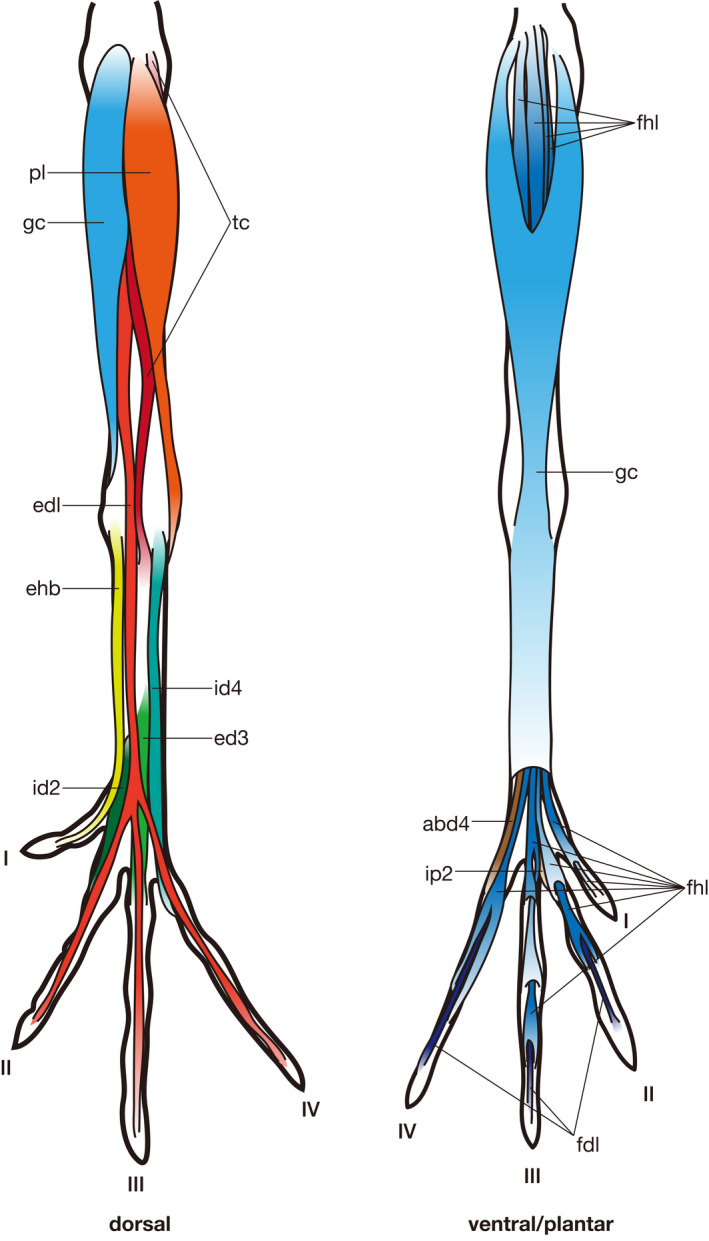
Pedal muscles of *Gallus gallu*s (modified after Yasuda, 2002). Some muscles are omitted for simplicity. See Table 5 for abbreviations

In the following section, the morphology of putatively homologous muscles across sauropsid clades is described under each heading of the proposed standardized muscle name. In order to clearly distinguish results of our first‐hand dissections and observations and descriptions cited from previous studies in the following descriptions, the former are provided in the past tense, whereas the latter are in the present tense followed by citations. Attachments on bones and superficial morphology of the described muscles are illustrated in figures: Figures 2 and 3 for Lepidodsauria, Figures 4 and 5 for Testudines, Figures 6 and 7 for Crocodilia, and Figures 8 and 9 for Aves. Homologies of muscles proposed in the present study were summarized in Table 5. Osteological correlates were recognized in more than 80% of examined muscles and were summarized in Table 6. Although the morphology of these muscles and associated osteological correlates were almost the same within each clade, there were a few differences recognized in this study as described below.

**Table 5:** Muscle homology and nomenclature adopted in the present study

Standardized muscle name	Abbreviation	Lepidosauria (Russell and Bauer, 2008)	Testudines (Walker, 1973)	Crocodilia (Suzuki *et al*., 2011)	Aves (Vanden Berge and Zweers, 1993)
M. tibialis cranialis	tc	M. extensor digitorum longus	M. extensor digitorum communis	M. extensor digitorum longus	M. tibialis cranialis
M. extensor digitorum longus	edl	M. tibialis anterior	M. tibialis anterior	M. tibialis anterior	M. extensor digitorum longus
M. peroneus longus	pl	M. peroneus longus	M. peroneus anterior	M. peroneus longus	M. fibularis longus
M. peroneus brevis	pb	M. peroneus brevis	M. peroneus posterior	M. peroneus brevis	M. fibularis brevis
M. adductor hallucis dorsalis	ahd	M. adductor et extensor hallucis et indicus	M. extensor hallucis proprius	M. adductor hallucis dorsalis	
M. extensor digitorum brevis	edb	Mm. extensores digitores breves (2, 3), in part	Mm. extensores digitorum breves	M. extensor digitorum I, II, et III	
M. extensor hallucis brevis	ehb	Mm. extensores digitores breves (1)	M. abductor hallucis	M. extensor hallucis brevis	M. extensor hallucis longus
M. extensor digiti II	ed2	Mm. extensores digitores breves (2), in part	Mm. interossei dorsales, in part	M. extensor digiti II	
M. extensor digiti III	ed3	Mm. extensores digitores breves (3), in part	Mm. interossei dorsales, in part	M. extensor digiti III	M. extensor brevis digiti III
M. extensor digiti IV	ed4	Mm. extensores digitores breves (4), in part	Mm. interossei dorsales, in part	M. extensor digiti IV	
M. interosseous dorsalis digiti II	id2	Mm. extensores digitores breves (2), in part	Mm. interossei dorsales, in part	M. interosseous dorsalis digiti II	M. abductor digiti II
M. interosseous dorsalis digiti III	id3	Mm. extensores digitores breves (3), in part	Mm. interossei dorsales, in part	M. interosseous dorsalis digiti III	
M. interosseous dorsalis digiti IV	id4	Mm. extensores digitores breves (4), in part	Mm. interossei dorsales, in part	M. interosseous dorsalis digiti IV	M. extensor brevis digiti IV
M. gastrocnemius	gc	M. gastrocnemius	M. gastrocnemius	M. gastrocnemius	M. gastrocnemius
M. flexor hallucis longus	fhl	M. flexor digitorum longus (femoral head)	M. flexor digitorum longus, in part	M. flexor digitorum longus	M. flexor hallucis longus M. flexor perforatus digiti II M. flexor perforatus digiti III M. flexor perforatus digiti IV M. flexor perforans et perforatus digiti II M. flexor perforans et perforatus digiti III
M. flexor digitorum longus	fdl	M. flexor digitorum longus (fibular head)	M. flexor digitorum longus, in part	M. flexor hallucis longus	M. flexor digitorum longus M. plantaris
M. pronator profundus	pp	M. pronator profundus	M. pronator profundus	M. pronator profundus	
M. fibulocalcaneous	fc	M. abductor digiti quinti		M. fibulocalcaneous	
M. flexor digitorum brevis superficialis	fdbs	M. flexores digitores breves +pfdl. calc.	Plantar aponeurosis	M. flexor digitorum brevis superficialis	
M. flexor digitorum brevis profundus	fdbp	pfdl. met. 5		M. flexor digitorum brevis profundus	
Mm. lumbricales	l	Mm. lumbricales	M. flexor digitorum communis sublimis	Mm. lumbricales	
M. lumbricalis profundus	lp	pfdl. ap.	Mm. lumbricales	M. lumbricalis profundus	M. lumbricalis
M. flexor hallucis brevis	fhb	M. flexor hallucis	Mm. interossei plantares, in part	M. flexor hallucis brevis superficialis M. flexor hallucis brevis profundus	
M. adductor digiti V	add5	M. adductor digiti quinti			
Mm. contrahentes	co	Mm. contrahentes	Mm. interossei plantares, in part	M. flexor digiti II M. flexor digiti III M. flexor digiti IV	
M. adductor hallucis plantaris	ahp	Mm. interossei plantales (1)	Mm. interossei plantares, in part	M. adductor hallucis plantaris	M. flexor hallucis brevis
M. interosseous plantaris digiti II	ip2	Mm. interossei dorsales, in part Mm. interossei plantares (2)	Mm. interossei plantares, in part	M. interosseous plantaris digiti II	M. adductor digiti II
M. interosseous plantaris digiti III	ip3	Mm. interossei dorsales, in part Mm. interossei plantares (3)	Mm. interossei plantares, in part	M. interosseous plantaris digiti III	
M. abductor digiti IV	abd4	Mm. extensores digitores breves (5)	Mm. interossei plantares, in part	M. abductor digiti IV dorsalis M. abductor digiti IV plantaris	M. abductor digiti IV

Arabic numerals in parentheses indicate the digits on which each muscle acts.

**Table 6:** List of osteological correlates of the origin and insertion of each muscle observed in the present study

Standardized muscle name	Origin/Insertion	Lepidosauria	Testudines	Crocodilia	Aves
M. tibialis cranialis	Origin	Absent	Absent	Absent	The lateral expansion of the cranial cnemial crest and the dorsoventral expansion of the lateral cnemial crest
	Insertion	A tubercle on each lateroplantar aspect of MTs II & III	A laterodorsal surface of each distal shaft of MTs I–IV	A tubercle on the medial margin of each proximal shaft of MTs II–IV	Tubercles on the dorsal aspect of the proximal tarsometatarsus (tuberositas m. tibialis cranialis)
M. extensor digitorum longus	Origin	Absent	A rugose longitudinal sulcus on the dorsomedial margin of the tibia	A rugose surface on the proximal‐most portion of the dorsal aspect of the tibia	A broad surface between the cranial and the lateral cnemial crests
	Insertion	Absent	A tubercle on the proximomedial end of MT I	A bulge on each dorsal aspect of MTs I and II	The proximodorsal lip of each phalanx of digits II–IV except proximal‐most phalanges
M. peroneus longus	Origin	Absent	A slightly excavated dorsal surface on the distal half of the fibula	A laterodorsal surface of the fibula, between each longitudinal ridge on the dorsal and the lateral aspects of the shaft	Proximal margins of the cranial and the lateral cnemial crests
	Insertion	A depression on the dorsolateral aspect of MT V	Absent	A flat surface on the proximolateral margin of MT V	Absent
M. peroneus brevis	Origin	Absent	Absent	A flat laterodorsal surface on the distal two thirds of the fibular shaft	A laterodorsal surface of the distal two thirds of the tibial shaft and a dorsal surface of the distal half of the fibular shaft
	Insertion	A process on the proximolateral margin of MT V (outer process)	Absent	Absent	A proximal surface near the lateral margin of the hypotarsus (tuberculum m. fibularis brevis)
M. adductor hallucis dorsalis	Origin	A dorsolateral surface of the distal end of the fibula and the medial half of the proximolateral lip of the tarsal facet of the astragalocalcaneum	A depression on the distodorsal margin of the tibia	Absent	NA
	Insertion	Absent	Absent	Absent	NA
M. extensor digitorum brevis	Origin	A depression on the dorsal aspect of astragalocalcaneum	A depression on the dorsal aspect of DT IV	A depression on the dorsal aspect of the astragalus	NA
	Insertion	The proximodorsal lip of each phalanx of digits III and IV (as well as digits I and II in some taxa)	The proximodorsal lip of each phalanx of digits III and IV	The proximodorsal lip of each phalanx of digits I–III	NA
M. extensor hallucis brevis	Origin	Absent	Absent	The dorsal aspect of MT I, distal to the transverse ridge	Longitudinal depressions on the medial parts of the dorsal aspects of the tarsometatarsus and MT I
	Insertion	The proximodorsal lip of each phalanx of digit I	The proximodorsal lip of I−2	The proximodorsal lip of each phalanx of digit I	The proximodorsal lip of each phalanx of digit I
M. extensor digiti II	Origin	Absent	Absent	Absent	NA
	Insertion	The proximodorsal lip of each phalanx of digit II	The proximodorsal lip of each of II−2 and 3	The proximodorsal lip of each phalanx of digit II	NA
M. extensor digiti III	Origin	Absent	Absent	Absent	A slightly depressed rugose surface on the dorsal aspect of the distal half of the tarsometatarsus
	Insertion	The proximodorsal lip of each phalanx of digit III	The proximodorsal lip of each of III−2, 3, and 4	The proximodorsal lip of each phalanx of digit III	The proximodorsal lip of III−1
M. extensor digiti IV	Origin	Lateral half of the proximolateral lip of the tarsal facet of the astragalocalcaneum	Absent	Absent	NA
	insertion	The proximodorsal lip of each of IV−2, 3, 4 and 5	The proximodorsal lip of each of IV−2, 3, 4 and 5	The proximodorsal lip of each phalanx of digit IV	NA
M. interosseous dorsalis digiti II	Origin	A longitudinal rugosity along the lateral margin of MT I	A rugosity on the proximomedial margin of the dorsal aspect of MT I	A depression on the lateroplantar aspect of MT I	An oblique groove on the medial aspect of the tarsometatarsus and the fossa metatarsi I
	Insertion	The proximodorsal lip of each phalanx of digit II	The proximodorsal lip of each phalanx of digit II	The proximodorsal lip of II−1	A medioplantar tubercle on the proximal end of II−1
M. interosseous dorsalis digiti III	Origin	A longitudinal rugosity along the lateral margin of the proximal half of MT II	A rugosity on the proximomedial margin of the dorsal aspect of MT II	A rugosity on the proximolateral margin of each of dorsal and lateral aspects of MT II	NA
	Insertion	The proximodorsal lip of each phalanx of digit III	The proximodorsal lip of each of III−2, 3, and 4	The proximodorsal lip of III−1	NA
M. interosseous dorsalis digiti IV	Origin	A longitudinal rugosity on the lateral margin of the proximal half of MT III	A rugosity on the proximomedial margin of the dorsal aspect of MT III	A depression on the lateroplantar aspect of the proximal shaft of MT III	A sulcus laterally along the origin of m. extensor brevis digiti III
	Insertion	The proximodorsal lip of each pahalnx of digit IV	The proximodorsal lip of each of IV−2 and 3	The proximodorsal lip of IV−1	A tuber on the medial aspect of IV−1
M. gastrocnemius	Origin	The medial and the lateral femoral epicondyles and distal portion of the tibial ventral crest	A rugose swelling on the medial margin of the dorsal aspect of the tibia, a blunt depression on the ventral aspect of the proximal tibia, and a depression on the lateroventral aspect of the distal femur	A depression on the lateroventral margin of the distal‐most portion of the shaft of the femur	A rugose depression on the lateroventral margin of the distal‐most portion of the femoral shaft, a shallow depression just proximal to the medial distal condyle of the femur, and the medial aspect of the cranial cnemial crest of the tibia
	Insertion	The medial and the lateral plantar tubercles of MT V	A prominent tubercle on the lateral margin of the plantar aspect of MT V	The lateral flange of MT V	Longitudinal ridges on the medial and lateral margins of the plantar aspect of the hypotarsus and the tarsometatarsal shaft
M. flexor hallucis longus	Origin	Absent	A shallow and broad depression on the lateroventral aspect of the femur	Absent	Several facets on the distal end of the fossa poplitea of the femur
	Insertion	The flexor tubercle of each ungual of digits II–IV	The flexor tubercle of each ungual of digits I–IV	The flexor tubercle of each ungual of digits I–IV	The flexor tubercle of I−2 and the proximoplantar heel of each non‐ungual phalanx of digits II–IV
M. flexor digitorum longus	Origin	A depression on the medial aspect of the fibula, just distal to the fibular attachment of m. popliteus	A distinct surface between the lateral margin of the shaft and the longitudinal ridge situated laterally on the ventral aspect of the fibula	A flat surface on the ventral aspect of the fibula, and depression on the ventral aspect of the proximal‐most tibial shaft	A flat surface on the dorsal aspect of the proximal one third and the lateral and ventral aspects of the distal two thirds of the fibular shaft
	Insertion	The flexor tubercle of each ungual of digits II–IV	The flexor tubercle of each ungual of digits I–IV	The flexor tubercle of each ungual of digits I–IV	The flexor tubercle of each ungual of digits II–IV
M. pronator profundus	Origin	A flat surface on the ventral aspect of fibula	A flat surface between two longitudinal ridges on the ventral aspect of the fibular shaft	A flat surface between longitudinal stout ridges on the dorsolateral and lateral margins on the ventral aspect of the tibial shaft	NA
	Insertion	Rugose surfaces or swellings on the proximomedial margins of the plantar aspects of MTs I–III	Absent	Narrow and rugose swelling on the proximomedial margin of the plantar aspect of MT I	NA
M. fibulocalcaneous	Origin	A smooth surface on the plantar aspect of the calcaneal tuber	NA	A flat surface on the ventral aspect of the distal one third of the fibula	NA
	Insertion	Absent	NA	The dorsal surface of the calcaneal tuber	NA
M. flexor digitorum brevis superficialis	Origin	Absent	Absent	Longitudinal ridge or bulge on the lateroplantar margin of MT V	NA
	Insertion	The proximoplantar heel of each non‐ungual phalanx of digits I–IV	The proximoplantar heel of each of II−1, III−1, and IV−1	The proximoplantar heel of each non‐ungual phalanx of digits I–III	NA
M. flexor digitorum brevis profundus	Origin	A concavity on the medial aspect of MT V	NA	Medial and lateral margins of the calcaneal tuber and the concavity on the distomedial margin of the plantar aspect of MT V	NA
	Insertion	The flexor tubercle of each ungual of digits I–IV	NA	The flexor tubercle of the unguals of digits I–III	NA
Mm. lumbricales	Origin	Absent	Absent	Absent	NA
	Insertion	The proximoplantar heel of each non‐ungual phalanx of digits III and IV	Absent	Absent	NA
M. lumbricalis profundus	Origin	Absent	Absent	Absent	Absent
	Insertion	The proximoplantar heel of each of II−1, III−1, and IV−1	Absent	Absent	Absent
M. flexor hallucis brevis	Origin	The plantar tubercle of DT IV	A depression on the plantar aspect of DT I	Absent	NA
	Insertion	The proximoplantar heel of I−1	The proximoplantar heel of I−1	The longitudinal depression on the planter surface of MT I and the proximoplantar heel of I−1	NA
M. adductor digiti V	Origin	Absent	NA	NA	NA
	Insertion	Absent	NA	NA	NA
Mm. contrahentes	Origin	A small tubercle on the medial margin of the shaft of MT V	Absent	A depression on each plantar aspect of DTs III and IV and a tubercle on the distal end of MT V	NA
	Insertion	Absent	Absent	The medioplantar surface of each of MTs II, III, and IV	NA
M. adductor hallucis plantaris	Origin	The dorsomedial surface of the proximal shaft of MT II	A depression on the medial aspect of the proximal portion of MT II	A depression on the mediodorsal aspect of near the proximal end of MT II	The medial part of the sulcus flexorius of the tarsometatarsus
	Insertion	Absent	Absent	A broad, shallow depression on the plantar aspect of MT I	The proximoplantar heel of I‐1
M. interosseous plantaris digiti II	Origin	The dorsomedial and medioplantar surfaces of the proximal shaft of MTIII	A depression on the medial aspect of MT III	Depressions on the dorsomedial and medioplantar aspects of near the proximal end of MT III	The central part of the distal sulcus flexorius
	Insertion	The lateroplantar surface MT II	Absent	The lateroplantar surface of MT II	A small tubercle on the lateroplantar margin of the proximal end of II−1
M. interosseous plantaris digiti III	Origin	The medial and the plantar surfaces of proximal two thirds of MT IV	A depression on the medial aspect of MT IV	Depressions on the dorsomedial and medioplantar aspects near the proximal end of MT IV	NA
	Insertion	The lateroplantar surface of MT III	Absent	The lateroplantar surface of MT III	NA
M. abductor digiti IV	Origin	The dorsomedial surface of MT V	A depression on the distal aspect of the medial portion of MT V	The mediodistal margin of the dorsal aspect of MT V	The lateral portion of the sulcus flexorius
	Insertion	Absent	Absent	A flat surface between the plantar ridge and the medial margin of MT IV	A tubercle projecting plantarly on the lateroproximal margin of IV−1

NA indicates the absence of the muscle itself.

### 3.1 Dorsal Musculature

#### 3.1.1 Musculus tibialis cranialis

Lepidosauria: M. extensor digitorum longus (Russell and Bauer, 2008)
Testudines: M. extensor digitorum communis (Walker, 1973)
Crocodilia: M. extensor digitorum longus (Suzuki *et al*., 2011)
Aves: M. tibialis cranialis (Vanden Berge and Zweers, 1993)

*Lepidosauria*: In the dissected specimens of *Iguana* and *Varanus*, m. extensor digitorum longus arose from the dorsomedial aspect of the lateral distal condyle of the femur and inserted on the metatarsals (MTs) II and III by two tendons, as described by Russell and Bauer (2008). Each insertion tendon passed the lateral aspect of the proximal shaft of each of MTs II and III, and inserted on the tubercle on its lateroplantar aspect. It was innervated by the fibular nerve.

*Testudines*: In the examined specimen of *Chelydra*, m. extensor digitorum communis arose from the dorsal aspect of the lateral distal condyle of the femur and inserted on the lateral sides of MTs I–IV and the proximodorsal lip of I‐2, as seen in *Emys* (Ribbing, 1938). Among testudines, there are some variations in the insertion site. For example, Gadow (1882) described that this muscle inserts on the proximal phalanges of digits I–IV. This muscle is innervated by the fibular nerve (Walker, 1973).

*Crocodilia*: In the examined specimens of *Paleosuchus* and *Crocodylus*, m. extensor digitorum longus arose from the dorsal aspect of the femur, just proximal to the lateral distal condyle, as described in previous studies (Gadow, 1882; Cong *et al*., 1998; Suzuki *et al*., 2011). It inserted on the tubercle on the medial margin of each proximal shaft of MTs II–IV, whereas an additional insertion on MT I is reported by Gadow (1882), Ribbing (1909, 1938) and Cong *et al*. (1998). On the medial‐most insertion site, the muscle merged with m. tibialis anterior as described by Suzuki *et al*. (2011). This muscle is innervated by the fibular nerve (Gadow, 1882; Ribbing, 1909, 1938; Cong *et al*., 1998; Suzuki *et al*., 2011).

*Aves*: In the dissected specimens of *Gallus* and *Grus*, m. tibialis cranialis took the origin on the distal extremity of the lateral distal condyle of the femur (fovea tendinis m. tibialis cranialis; Vanden Berge and Zweers, 1993) and the cranial and lateral cnemial crests of the tibia (Fujioka, 1962; Vanden Berge, 1975). The tibial origins were on the lateral expansion of the cranial cnemial crest and the dorsoventral expansion of the lateral cnemial crest. This muscle inserted on the tubercles on the dorsal aspect of the proximal tarsometatarsus (tuberositas m. tibialis cranialis; Vanden Berge and Zweers, 1993). It is innervated by the common fibular nerve (Vanden Berge, 1975).

*Comparison*: Except for the additional origin on the proximal extremity of the tibia in avians, the sites of origin on the femur are conserved among sauropsids. The general innervation pattern also remains conservative. The insertion is generally on MTs II and III, although additional insertions are variably present. The insertion scar on the dorsal aspect of the tarsometatarsus in avians, the tuberositas m. tibialis cranialis, appears to have been originally situated on MTs II and III before the fusion among MTs. Although the name meaning a digital extensor is commonly used for this muscle in non‐avian sauropsids, the avian name m. tibialis cranialis is used to indicate this and all other sauropsid homologues in the following discussion.

#### 3.1.2 Musculus extensor digitorum longus

Lepidosauria: M. tibialis anterior (Russell and Bauer, 2008)
Testudines: M. tibialis anterior (Walker, 1973)
Crocodilia: M. tibialis anterior (Suzuki *et al*., 2011)
Aves: M. extensor digitorum longus (Vanden Berge and Zweers, 1993)

*Lepidosauria*: In the dissected specimens of *Iguana* and *Varanus*, m. tibialis anterior took the origin from the broad area on the shaft of the tibia. In *Iguana*, the origin occupied the proximal two thirds on the dorsal surface and the distal two thirds on the ventral aspect of the shaft of the tibia. In *Varanus*, in contrast, the origin on the dorsal surface continues toward distal end of the tibia. The insertion was on the medial margin of MT I, as described by Russell and Bauer (2008). It was innervated by the fibular nerve.

*Testudines*: In the examined *Chelydra*, m. tibialis anterior arose from the dorsomedial margin of the tibia and inserted on the proximomedial end of MT I, as described by Walker (1973). The origin and insertion on bones were marked by a rugose, longitudinal sulcus, and a tubercle, respectively. It is innervated by the fibular nerve (Walker, 1973).

*Crocodilia*: In *Paleosuchus* and *Crocodylus* dissected in the present study, m. tibialis anterior arose from the proximal‐most portion of the dorsal aspect of the tibia marked with surface rugosity and inserted on the bulge on the dorsal aspect of each of MTs I and II. Laterally, the area of origin became broader proximodistally and was marked by distinct rugosity on the bone, as described by Suzuki *et al*. (2011). This muscle is innervated by the fibular nerve (Gadow, 1882; Cong *et al*., 1998; Suzuki *et al*., 2011).

*Aves*: In the examined *Gallus* and *Grus*, m. extensor digitorum longus took the origin from the broad surface between the cranial and lateral cnemial crests and the dorsolateral aspect of the tibial shaft. The area of origin tapered distally. It inserted on processes on the proximodorsal lips of all phalanges of digits II‐IV except for the most proximal phalanges (also described by Fujioka, 1962; Vanden Berge, 1975). This muscle is innervated by the common fibular nerve (Vanden Berge, 1975).

*Comparison*: The origin and innervation pattern of this muscle are conserved among sauropsids. However, the insertion is different between avians (on the distal phalanges) and non‐avian sauropsids (generally on MT I with some additional areas). The homology between non‐avian sauropsids and avians proposed here has also been considered, but not adopted, by Hutchinson (2002). Although the name ‘m. tibialis anterior’ is used in the majority of studies on sauropsids, the avian ‘m. extensor digitorum longus’ is adopted here as the general name to cleary indicate that this muscle is different from otherwise similarly named m. tibialis cranialis discussed above.

#### 3.1.3 Musculus peroneus longus

Lepidosauria: M. peroneus longus (Russell and Bauer, 2008)
Testudines: M. peroneus anterior (Walker, 1973)
Crocodilia: M. peroneus longus (Suzuki *et al*., 2011)
Aves: M. fibularis longus (Vanden Berge and Zweers, 1993)

*Lepidosauria*: M. peroneus longus takes its origin from the lateral femoral epicondyle, just distal to the origin of m. flexor digitorum longus (Russell and Bauer, 2008). In the examined *Iguana* and *Varanus*, the insertion was marked by a depression on the dorsolateral aspect of the shaft of MT V (contra “lateral plantar tubercle” in Russell and Bauer, 2008). It is innervated by the fibular nerve (Jullien, 1967).

*Testudines*: In the examined *Chelydra*, m. peroneus anterior arose from the distal half of the dorsal aspect of the fibula and inserted on the dorsal aspect of the proximolateral margins of MT V and IV‐1. The site of origin on the lateral fibula was marked by a slightly excavated surface. It is innervated by the fibular nerve (Walker, 1973).

*Crocodilia*: In the dissected specimens of *Paleosuchus* and *Crocodylus*, m. peroneus longus arose from an almost entire laterodorsal aspect of the fibula (also described by Suzuki *et al*., 2011). This area of origin was the surface between longitudinal ridges on the dorsal and the lateral aspects of the bone. This muscle partly merged with m. peroneus brevis and inserted on the flat surface on the proximolateral margin of MT V, distally adjacent to a separate insertion site of m. peroneus brevis. This muscle is innervated by the superficial fibular nerve (Cong *et al*., 1998; Suzuki *et al*., 2011).

*Aves*: In *Gallus*, this muscle arises from the proximal margins of the cranial and lateral cnemial crests, the patellar tendon, and associated fascia (Fujioka, 1962). In *Eudromia*, the origin extends on the lateral aspect of the anterior cnemial crest (Suzuki *et al*., 2014). It inserts on soft tissues, sustentaculum tarsi, and the insertion tendon of the flexor perforatus digiti III (Fujioka, 1962; Vanden Berge, 1975). This muscle is innervated by the fibular nerve (Vanden Berge, 1975).

*Comparison*: The homology of this muscle hypothesized in the present study follows the one proposed by Dilkes (2000) and Hutchinson (2002). The areas of origin in crocodilians and testudines are similar to each other, but differ from those in both lepidosaurs and avians. The insertion is conserved among non‐avians, whereas the one in avians is different possibly because of the loss of MT V.

#### 3.1.4 Musculus peroneus brevis

Lepidosauria: M. peroneus brevis (Russell and Bauer, 2008)
Testudines: M. peroneus posterior (Walker, 1973)
Crocodilia: M. peroneus brevis (Suzuki *et al*., 2011)
Aves: M. fibularis brevis (Vanden Berge and Zweers, 1993)

*Lepidosauria*: M. peroneus brevis takes its origin on the almost entire anterior, lateral and medial surfaces of the fibula for almost its entire length, and inserts on the process on the proximolateral margin of MT V (outer process; Russell and Bauer, 2008). This muscle is innervated by the fibular nerve, as is m. peroneus longus (Jullien, 1967).

*Testudines*: M. peroneus brevis arises from the distal end of the fibula and inserts on the proximal margin of the dorsal aspect of MT V, thus, acting with m. peroneus longus to extend and abduct digit V and probably also assisting in dorsiflexion of the pes (Walker, 1973). This muscle is innervated by the fibular nerve (Ribbing, 1938).

*Crocodilia*: In the dissected *Paleosuchus* and *Crocodylus*, m. peroneus brevis arose from the laterodorsal aspect of the distal two thirds of the shaft of the fibula and inserted on the proximolateral margin of MT V, as described by Cong *et al*. (1998) and Suzuki *et al*. (2011). The origin was a flat surface. It is innervated by the superficial fibular nerve (Cong *et al*., 1998; Suzuki *et al*., 2011).

*Aves*: M. fibularis brevis mainly arises from the most of the interosseal space between the tibia and the fibula (Fujioka, 1962; Vanden Berge, 1975; Yasuda, 2002) and inserts on a proximal surface near the lateral margin of the hypotarsus (tuberculum m. fibularis brevis; Vanden Berge and Zweers, 1993). The tibial origin was a narrow surface between two longitudinal ridges on the distal two thirds of the shaft, whereas the fibular origin was the dorsal surface of the distal half of the shaft. This muscle is innervated by the deep fibular nerve (Vanden Berge, 1975).

*Comparison*: Although the innervation pattern is different between crocodilians and avians, the homology of these muscles proposed here was also discussed and supported by Hutchinson (2002). The innervation pattern is conserved among sauropsids, except between crocodilians and avians, in which the superficial and deep branches of the fibular nerve supply the muscle, respectively.

#### 3.1.5 Musculus adductor hallucis dorsalis

Lepidosauria: M. adductor et extensor hallucis et indicus (Russell and Bauer, 2008)
Testudines: M. extensor hallucis proprius (Walker, 1973)
Crocodilia: M. adductor hallucis dorsalis (Suzuki *et al*., 2011)

*Lepidosauria*: In the examined *Iguana*, m. adductor et extensor hallucis et indicus arose from a broad and flat or slightly depressed surface on the dorsolateral aspect of the distal fibula as well as from the medial half of a slight convex lip at the proximolateral margin of the tarsal facet of the astragalocalcaneum. This muscle inserted on the medial margin and the laterodistal part of the shaft of MT I as well as on a small area on the proximodorsal aspect of the shaft of MT II, as described by Russell and Bauer (2008). The insertion area is more limited in some taxa such as *Varanus* and *Gekko* (only on MT I) and *Chamaeleo* (only on MT II; Russell and Bauer, 2008). *Varanus* showed rugosity at the insertion area on the bone. The deep fibular nerve innervates this muscle (Ribbing, 1938:fig. 562).

*Testudines*: In the dissected specimen of *Chelydra*, m. extensor hallucis proprius arose from the distal end of the fibula and the dorsal aspect of the astragalocalcaneum, and the origin on the fibula was marked as a clear depression on the distodorsal margin of the bone. It distally merged with m. abductor hallucis and inserted on the lateral sides of the distal one third of MT I and the proximal half of I‐1 as well as on the medial side of the mid‐shaft of I‐1. In *Trachemys*, the origin on the astragalocalcaneum is absent (Walker, 1973, fig. 22). This muscle is innervated by the deep fibular nerve (Ribbing, 1938, fig. 560).

*Crocodilia*: In the examined *Paleosuchus* and *Crocodylus*, m. adductor hallucis dorsalis arose from the dorsal aspect of the distal fibula and inserted on the dorsal aspect of the proximal one third of the shaft of MT I, just proximal to the insertion of ‘m. tibialis anterior.’ In *Crocodylus*, the insertion has previously been described as divided into two sites (Suzuki *et al*., 2011). The presence of such two separate insertions, however, was not confirmed in the same species dissected in this study, thus, suggesting a possible intraspecific variation. The distal border of the insertion of this muscle was marked by a transverse ridge on the dorsal aspect of MT I. This muscle is innervated by the fibular nerve (Gadow, 1882; Ribbing, 1938; Cong *et al*., 1998; Suzuki *et al*., 2011).

*Aves*: This muscle is apparently absent in avians.

*Comparison*: The sites of origin and insertion and the innervation pattern are almost completely conserved among non‐avian sauropsid taxa. The homology of this muscle among sauropsids has already been discussed by Gadow (1882). Although Hutchinson (2002) regard this muscle as a homologue of m. extensor hallucis longus in avians (*sensu* Vanden Berge and Zweers, 1993), such a homology hypothesis is not accepted in this study (see the description of m. extensor hallucis brevis below).

#### 3.1.6 Musculus extensor digitorum brevis

Lepidosauria: Mm. extensores digitores breves, in part (Russell and Bauer, 2008)
Testudines: Mm. extensores digitorum breves (Walker, 1973)
Crocodilia: M. extensor digitorum I, II et III (Suzuki *et al*., 2011)

*Lepidosauria*: In *Iguana* dissected in the present study, two proximal heads of mm. extensores digitores breves arose from the dorsal depression on the astragalus and inserted on the proximodorsal lip of each phalanx of digits III and IV. These slips have been described as two portions of mm. extensores breves (Russell and Bauer, 2008). In *Varanus* dissected in the present study, additional two slips inserting on the proximodorsal lips of digits I and II were present. This muscle is innervated by the deep fibular nerve (Ribbing, 1938).

*Testudines*: In the examined *Chelydra*, mm. extensores digitorum breves arose from the depression on the dorsal aspect of distal tarsal (DT) IV, and distally merged with mm. interossei dorsales, which together inserted on the proximodorsal lips of distal phalanges of digits III and IV. In *Trachemys*, this muscle has an additional origin from DT III and insertion on digit II (Walker, 1973). This muscle is innervated by the fibular nerve (Ribbing, 1938).

*Crocodilia*: In the dissected specimens of *Paleosuchus* and *Crocodylus*, m. extensor digitorum I, II et III arose from the depression on the dorsal aspect of the astragalus and inserted dorsally on digits I–III by the dorsal aponeurosis. In *Caiman*, however, the origin is not on the astragalus but on the soft tissue between this bone and the calcaneum (Suzuki *et al*., 2011). This muscle is innervated by the deep fibular nerve (Gadow, 1882; Cong *et al*., 1998; Suzuki *et al*., 2011).

*Aves*: This muscle is apparently absent in avians.

*Comparison*: The concentrated origin on the dorsal aspect of the tarsus is conserved among non‐avian sauropsid taxa. Between lepidosaurs and crocodilians, although the bellies inserting on digits I and II are sometimes absent in the former (at least in *Iguana*) and the one on digit IV is absent in the latter, the similarity in the concentrated origin on the astragalus and its osteological correlation suggests the homologous relationship between the muscles described here. Although the origin in turtles is slightly different from these lepidosaurian and crocodilian muscles, homology of this muscle among these taxa was also supported by Gadow (1882). Although this muscle has been distinguished from other short extensors in crocodilians as m. extensor digitorum I, II et III by Suzuki *et al*. (2011), the name m. extensor digitorum brevis is proposed as the standard name for this and all homologous muscles because these muscles sometimes lack insertions on digits I–II and/or gains the one on digit IV in non‐crocodilian sauropsids.

#### 3.1.7 Musculus extensor hallucis brevis

Lepidosauria: Mm. extensores digitores breves, in part (Russell and Bauer, 2008)
Testudines: M. abductor hallucis (Walker, 1973)
Crocodilia: M. extensor hallucis brevis (Suzuki *et al*., 2011)
Aves: M. extensor hallucis longus (Vanden Berge and Zweers, 1993)

*Lepidosauria*,*Testudines*,*and Crocodilia*: The morphology of this muscle was mostly conserved among non‐avian sauropsids. In the examined leidosaurs and crocodilians, it took the main origin from the dorsal aspect of MT I and inserted on the proximodorsal lips of I‐1 and I‐2, as described by Cong *et al*. (1998) and Suzuki *et al*. (2011). It is innervated by the deep fibular nerve (Ribbing, 1938; Russell and Bauer, 2008; Suzuki *et al*., 2011). In testudines, m. abductor hallucis has an additional origin on DT I (in *Trachemys*; Walker, 1973). In the examined *Chelydra*, it instead has the additional origin on I‐1. The innervation is by the deep fibular nerve as in lepidosaurs and crocodilians (Ribbing, 1938, fig. 560).

*Aves*: In the dissected specimens of *Gallus* and *Grus*, m. extensor hallucis longus originated from the medial portion of the sulcus extensorius and inserted on the proximodorsal lips of I‐1 and I‐2, as described by Fujioka (1962). An additional fleshy origin on the dorsal aspect of MT I was also observed. Both origins were marked by longitudinal depressions on the medial parts of the dorsal aspects of the tarsometatarsus and MT I. This muscle is innervated by the deep fibular nerve (Vanden Berge, 1975).

*Comparison*: Although the area of origin of the avian m. extensor hallucis longus is different from that of m. extensor hallucis brevis in non‐avian sauropsids, these muscles are the most similar in the site of insertion and innervation patterns, suggesting their homologous relationships.

#### 3.1.8 Musculus extensor digiti II

Lepidosauria: Mm. extensores digitores breves, in part (Russell and Bauer, 2008)
Testudines: Mm. interossei dorsales, in part (Walker, 1973)
Crocodilia: M. extensor digiti II (Suzuki *et al*., 2011)
*Lepidosauria*: In the dissected *Iguana* and *Varanus*, the slip of m. extensores digitores breves for digit II arose from the dorsal aspect of MT II and inserted on the proximodorsal lip of each phalanx of digit II, as described by Russell and Bauer (2008). This muscle is innervated by the deep fibular nerve (Ribbing, 1938)

*Testudines*: In the examined *Chelydra*, a portion of mm. interossei dorsales arose from the dorsal aspects of astragalocalcaneum, DT I, MT II, and II‐1, and inserted on the proximodorsal lips of distal phalanges of digit II. This muscle is the most likely to correspond to a portion of mm. interossei dorsales described by Walker (1973), although the origin of the latter does not attach on II‐1. It is innervated by the deep fibular nerve (Ribbing, 1938).

*Crocodilia*: In the dissected *Paleosuchus* and *Crocodylus*, m. extensor digiti II arose from the dorsal aspect of MT II and inserted on the proximodorsal lip of each phalanx of digit II, as described by Suzuki *et al*. (2011). This muscle is innervated by the deep fibular nerve (Ribbing, 1938; Cong *et al*., 1998; Suzuki *et al*., 2011).

*Aves*: This muscle is apparently absent in avians.

*Comparison*: The origin from the dorsal aspect of MT II and the innervation pattern are both conserved among non‐avian sauropsids. The additional origin on II‐1 is characteristic of Testudines but can be regarded as an expansion of the MT II origin. Although an additional origin on the proximal end of MT III was described by Russell and Bauer (2008) for the slip of mm. extensores digitores breves inserting on digit II in lepidosaurs, this head is recognized as a homologue of m. interosseous dorsalis digiti II in the present study.

#### 3.1.9 Musculus extensor digiti III

Lepidosauria: Mm. extensores digitores breves, in part (Russell and Bauer, 2008)
Testudines: Mm. interossei dorsales, in part (Walker, 1973)
Crocodilia: M. extensor digiti III (Suzuki *et al*., 2011)
Aves: M. extensor brevis digiti III (Vanden Berge and Zweers, 1993)

*Lepidosauria*: In the dissected specimens of *Iguana* and *Varanus*, a slip of mm. extensores digitores breves arose from the dorsolateral surface of the MT III shaft, as described by Russell and Bauer (2008). This muscle distally merged with the portions of m. extensor digitorum brevis and m. interosseous dorsalis digiti III (see below), and inserted on the proximodorsal lip of each phalanx of digit III. This muscle is supplied by the deep fibular nerve (Ribbing, 1938). Although Russell and Bauer (2008) described additional origins from the lateral aspect of the distal portion of MT II and the dorsal aspect of the distal one half of MT IV, such origins were not confirmed in the present study.

*Testudines*: In the examined *Chelydra*, a portion of mm. interossei dorsales mainly arose from the dorsal aspect of MT III with additional origins on DT III and III‐1. It inserted on the distal phalanges of digit III by a dorsal aponeurosis. In *Trachemys*, the origin on III‐1 and the insertion on III‐2 are absent (Walker, 1973). This muscle is supplied by the deep fibular nerve (Ribbing, 1938).

*Crocodilia*: In the dissected *Paleosuchus* and *Crocodylus*, m. extensor digiti III arose from the dorsal surface of MT III and inserted on the proximodorsal lip of each phalanx of digit III by a dorsal aponeurosis. There are some additional origins on the proximolateral margin of MT II in *Caiman* (Suzuki *et al*., 2011) and dorsal aspect of DT III in *Alligator* (Cong *et al*., 1998). Although Cong *et al*. (1998) reported an additional origin arising from the astragalus that is shared with M. extensor digiti II, this slip is here regarded as a part of m. extensor digiti I, II et III following Suzuki *et al*. (2011). This muscle is innervated by the fibular nerve (Gadow, 1882; Ribbing, 1938; Cong *et al*., 1998; Suzuki *et al*., 2011).

*Aves*: The possible homologue of the crocodilian m. extensor digiti III in *Gallus* and *Grus* arose from the dorsal aspect of the distal half of the tarsometatarsus and inserted on the proximodorsal lip of III‐1. In the examined *Gallus*, the origin was marked by a slightly depressed rugose surface. This muscle is innervated by the superficial fibular nerve (Vanden Berge, 1975).

*Comparison*: The origin and insertions of these muscles are mostly conserved among sauropsids except for the presence of an additional origin in Testudines and concentration of the insertion in Aves. These muscles are consistently supplied by the deep fibular nerve in lepidosaurs, testudines, and crocodilians and appear to be homologous among these clades (Ribbing, 1938).

#### 3.1.10 Musculus extensor digiti IV

Lepidosauria: Mm. extensores digitores breves, in part (Russell and Bauer, 2008)
Testudines: Mm. interossei dorsales, in part (Walker, 1973)
Crocodilia: M. extensor digiti IV (Suzuki *et al*., 2011)

*Lepidosauria*: In the examined *Iguana*, a portion of mm. extensores digitores breves, i.e., a slip arising from the lateral half of a slight convex lip at the proximolateral margin of the tarsal facet of the astragalocalcaneum, was joined on its deeper surface by some fibers of a slip arising from the dorsolateral (or “mesial” in Russell and Bauer, 2008) aspect of MT IV. A single tendon inserted on the proximodorsal lip of the ungual of digit IV, with accessory insertions on the proximal ends of IV‐2, IV‐3, and IV‐4. This muscle is innervated by the superficial fibular nerve (Ribbing, 1938).

*Testudines*: In the dissected *Chelydra*, a portion of mm. interossei dorsales had the main origin from the dorsal aspect of MT IV and additional origins from DT IV and IV‐1 and inserted on the distal phalanges of digit IV by a dorsal aponeurosis. In *Trachemys*, origin from IV‐1 and insertion on IV‐2 are absent (Walker, 1973). This muscle is innervated by the fibular nerve (Ribbing, 1938; Walker, 1973).

*Crocodilia*: In the dissected specimens of *Paleosuchus* and *Crocodylus*, m. extensor digiti IV arose from the dorsal surface of MT IV and inserted on the proximodorsal lip of each phalanx of the digit IV by a dorsal aponeurosis. Additional origins on the dorsal aspect of DT IV and the connective tissue between the calcaneum and DT IV have been described in *Alligator* (Cong *et al*., 1998) and *Caiman* (Suzuki *et al*., 2011), respectively. This muscle is innervated by the superficial fibular nerve in *Caiman* (Suzuki *et al*., 2011) and by the deep fibular nerve in *Alligator* (Cong *et al*., 1998).

*Aves*: This muscle is apparently absent in avians.

*Comparison*: All of these muscles in Lepidosauria, Testudines, and Crocodilia commonly arise from the dorsal aspect of MT IV, insert dorsally on the phalanges of digit IV, and are innervated by the fibular nerve. In addition, each of these muscles has one or more additional origins such as proximal heads from the tarsus or soft tissues.

#### 3.1.11 Musculus interosseous dorsalis digiti II

Lepidosauria: Mm. extensores digitores breves, in part (Russell and Bauer, 2008)
Testudines: Mm. interossei dorsales, in part (Walker, 1973)
Crocodilia: M. interosseous dorsalis digiti II (Suzuki *et al*., 2011)
Aves: M. abductor digiti II (Vanden Berge and Zweers, 1993)

*Lepidosauria*: In the examined *Iguana* and *Varanus*, a part of mm. extensores digitores breves arose from the ridge‐like, longitudinal rugose area along the lateral margin of MT I by a stout tendon, and inserted on the proximodorsal lip of each phalanx of digit II by a dorsal aponeurosis. In *Iguana*, the origin tendon extended along this muscle and joined the medial portion of the aponeurosis, which is regarded as an “oblique intermetatarsal ligament” by Russell and Bauer (2008). In *Varanus*, this tendon inserted on the medial margin of the distal portion of MT II, just proximal to the distal condyle. This muscle is innervated by the deep fibular nerve (Ribbing, 1938).

*Testudines*: In the dissected specimen of *Chelydra*, there was a slip arising from the rugosity on the proximomedial margin of the dorsal aspect of MT I. This slip merged with the main slip of mm. interossei dorsales extending to digit II and inserted on the distal phalanges of digit II by a dorsal aponeurosis. This muscle is innervated by the fibular nerve (Walker, 1973).

*Crocodilia*: In the dissected *Paleosuchus* and *Crocodylus*, m. interosseous dorsalis digiti II arose from the depression on the lateroplantar aspect of MT I, accompanied by a stout tendinous structure. The insertion tendon of this muscle joined the dorsal aponeurosis of digit II, but most of its fibers inserted on the proximodorsal lip of II‐1, as described by Suzuki *et al*. (2011). This muscle is innervated by the deep fibular nerve (Gadow, 1882; Suzuki *et al*., 2011).

*Aves*: In the examined specimens of *Gallus* and *Grus*, m. abductor digiti II arose from both the lateral aspect of MT I and the dorsomedial aspect of the tarsometatarsus and inserted on the medioplantar tubercle at the proximal end of II‐1. The origin on the tarsometatarsus was marked by an oblique groove on the medial aspect of the shaft and the fossa metatarsi I. This muscle is innervated by the deep fibular nerve (Vanden Berge, 1975).

*Comparison*: The muscles described above all share their origin on MT I, insert on digit II, and are innervated by the deep fibular nerve. These conserved patterns suggest these are homologous with one another. The association of this muscle with the ligament‐like structure in both Lepidosauria and Crocodilia also supports this hypothesis.

#### 3.1.12 Musculus interosseous dorsalis digiti III

Lepidosauria: Mm. extensores digitores breves, in part (Russell and Bauer, 2008)
Testudines: Mm. interossei dorsales, in part (Walker, 1973)
Crocodilia: M. interosseous dorsalis digiti III (Suzuki *et al*., 2011)

*Lepidosauria*: In the dissected *Iguana* and *Varanus*, a slip of mm. extensores digitores breves arose tendinously from the ridge‐like, longitudinal rugose area along the lateral margin of the proximal half of MT II. Distally, this muscle forms the medial portion of the dorsal aponeurosis of digit III, which inserted on the proximodorsal lip of each phalanx. In *Varanus*, an additional tendinous insertion was present on the medial margin of the distal portion of MT III, just proximal to the distal condyle. The belly of mm. extensores digitores breves for digit III described as “arising from the lateral aspect of the distal portion of the shaft of MT II” by Russell and Bauer (2008) probably corresponds to this muscle and represents a possible intraspecific variation. This muscle is innervated by the deep fibular nerve (Ribbing, 1938).

*Testudines*: In the examined specimen of *Chelydra*, one of several heads of mm. interossei dorsales arose from the rugosity on the proximomedial margin of the dorsal aspect of MT II and inserted on the proximodorsal lips of distal phalanges of digit III by a dorsal aponeurosis. This muscle is innervated by the fibular nerve (Walker, 1973).

*Crocodilia*: In the dissected *Paleosuchus* and *Crocodylus*, m. interosseous dorsalis digiti III arose from the rugose surface on the dorsal and lateral aspects of the lateral margin of proximal MT II. The insertion tendon of this muscle joined the dorsal aponeurosis of digit III, but most of its fibers inserted on the proximodorsal lip of III‐1, as described by Suzuki *et al*. (2011). This muscle is innervated by the fibular nerve (Gadow, 1882; Suzuki *et al*., 2011).

*Aves*: This muscle is apparently absent in avians.

*Comparison*: Although m. extensor digiti brevis III in avians has an insertion similar to those of the non‐avian muscles listed here, the former avian muscle is not regarded as the homologue of m. interosseous dorsalis digiti III in the present study. This is mainly because of the origin site of this avian muscle on the middle of the distal half of the tarsometatarsus more likely corresponds to MT III, rather than MT II from which m. interosseous dorsalis digiti III in non‐avians arises. In addition, this muscle in avian does not act as an adductor of digit III unlike m. interosseous digiti III in crocodilians (Suzuki *et al*., 2011) but instead is an extensor of this digit (Vanden Berge, 1975).

#### 3.1.13 Musculus interosseous dorsalis digiti IV

Lepidosauria: Mm. extensores digitores breves, in part (Russell and Bauer, 2008)
Testudines: Mm. interossei dorsales, in part (Walker, 1973)
Crocodilia: M. interosseous dorsalis digiti IV (Suzuki *et al*., 2011)
Aves: M. extensor brevis digiti IV (Vanden Berge and Zweers, 1993)

*Lepidosauria*: In the dissected *Iguana*, a portion of mm. extensores digitores breves arose from the rugose, longitudinal surface on the lateral margin of the proximal half of the shaft of MT III, and inserted medially on the proximal margin of each phalanx of digit IV as a medial portion of the dorsal aponeurosis. This muscle is innervated by the deep fibular nerve (Ribbing, 1938).

*Testudines*: In the examined *Chelydra*, mm. interossei dorsalis had an origin on the rugosity on the proximomedial margin of the dorsal aspect of MT III and inserted on the proximodorsal lips of distal phalanges of digit IV by a dorsal aponeurosis. This muscle is innervated by the fibular nerve (Walker, 1973).

*Crocodilia*: In the examined specimens of *Paleosuchus* and *Crocodylus*, m. interosseous dorsalis digiti IV arose from the depression on the lateroplantar aspect of the proximal‐most shaft of MT III. The insertion tendon of this muscle joined the dorsal aponeurosis of digit IV, but most of its fibers inserted on the proximodorsal lip of IV‐1, as described by Suzuki *et al*. (2011). This muscle is innervated by the fibular nerve (Suzuki *et al*., 2011). This muscle was not described in Cong *et al*. (1998) and thus is possibly absent in *Alligator sinensis*.

*Aves*: In the dissected *Gallus* and *Grus*, m. extensor brevis digiti IV arose from the dorsal aspect of the tarsometatarsus and inserted on the medial aspect of IV‐1, as described by Fujioka (1962). The course of the insertion tendon was marked by the canalis interosseus distalis (Figure 7; Vanden Berge and Zweers, 1993) situated proximal to the incisura intertrochlearis lateralis. The origin was marked by a sulcus laterally along the origin of m. extensor brevis digiti III, whereas the insertion was marked by a tuber. This muscle is innervated by the superficial fibular nerve (Vanden Berge, 1975).

*Comparison*: The origin from MT III is conserved between crocodilians and lepidosaurs. The identity or homology of the bone serving the origin for the avian muscle (i.e., MT III serves as the origin) is not certain, but the insertion on the medial aspect of IV‐1 is more similar to the insertion of m. interosseous dorsalis digiti IV than to that of m. extensor digiti IV, the latter of which arises from the dorsal aspect of MT IV and inserts dorsally on the phalanges of digit IV in crocodilians.

### 3.2 Plantar Musculature

#### 3.2.1 Musculus gastrocnemius

Lepidosauria: M. femorotibial gastrocnemius; M. femoral gastrocnemius (Russell and Bauer, 2008)
Testudines: M. gastrocnemius (Walker, 1973)
Crocodilia: M. gastrocnemius (Suzuki *et al*., 2011)
Aves: M. gastrocnemius (Vanden Berge and Zweers, 1993)

*Lepidosauria*: In the examined *Iguana*, m. femorotibial gastrocnemius arose as a complex of six tendons, two of which arose from the dorsomedial and ventromedial aspects of the medial femoral epicondyle, and one of which arose from the distal portion of the tibial ventral crest, as described by Russell and Bauer (2008). These slips became fused to form an aponeurosis, most of which distally merged with m. femoral gastrocnemius, m. flexor digitorum brevis superficialis, m. peroneus longus, and other small digital flexors. More distally, the remaining portion formed a narrow tendon to insert on the proximolateral margin of each phalanx of digit V, as described by Russell and Bauer (2008). In addition, this narrow tendon gave some fibers to m. abductor digiti IV. M. femoral gastrocnemius took its origin on the lateral epicondyle of the femur and additionally on the knee joint capsule and mainly inserted onto the medial and lateral plantar tubercles of MT V, as described by Russell and Bauer (2008). These muscles are innervated by the tibial nerve (Ribbing, 1938).

*Testudines*: In *Chelydra*, m. gastrocnemius had two heads, as described by Walker (1973). One of them arising from the proximal end of the tibia was further subdivided into two, the one arose from a rugose swelling on the medial margin of the dorsal aspect of the tibia and the other arose from a blunt depression on the ventral aspect of the proximal tibia. The other head took its origin from a depression on the lateroventral aspect of the distal femur. This muscle inserted on a prominent tubercle on the lateral margin of the plantar aspect of MT V. This muscle is innervated by the tibial nerve (Walker, 1973).

*Crocodilia*: In the dissected specimens of *Paleosuchus* and *Crocodylus*, the bony origin of m. gastrocnemius was on the lateroventral margin of the distal‐most portion of the shaft of the femur, and also on the medioventral aspect of the proximal half of the tibia, as described by Cong *et al*. (1998). The origin on the femur left a distinct depression. These bellies were distally fused and mainly inserted on the lateral flange of MT V, while giving some fibers to m. flexor digitorum brevis superficialis. This muscle is innervated by the tibial nerve (Gadow, 1882; Cong *et al*., 1998; Suzuki *et al*., 2011).

*Aves*: In the dissected *Gallus* and *Grus*, the lateral head (pars lateralis) of m. gastrocnemius arose from the lateroventral margin of the distal‐most portion of the femoral shaft, as described by Fujioka (1962) and Vanden Berge (1975), leaving a rugose depression. The intermediate head (pars intermedia) arose from the area just proximal to the medial distal condyle of the femur, as reported by the previous studies (Fujioka, 1962; Vanden Berge, 1975), leaving a shallow depression as its origin site. In *Eudromia*, however, this head arises from the lateral margin of the popliteal fossa (Suzuki *et al*., 2014). The medial head (pars medialis) arose from the medial aspect of the cranial cnemial crest of the tibia, with the origin extending onto the patellar tendon composed of m. femorotibialis medialis and/or m. iliotibialis cranialis, as described in the previous studies (Fujioka, 1962; Vanden Berge, 1975; Suzuki *et al*., 2014). The insertion of this muscle was on the medial and lateral margins of the plantar aspects of the hypotarsus and the shaft of tarsometatarsus, forming longitudinal ridges in the adult. The insertion tendon ossifies in some taxa (Vanden Berge, 1975). This muscle is innervated by the tibial nerve (Vanden Berge, 1975).

*Comparison*: These muscles commonly arise from the ventral aspect of the lateral distal condyle of the femur as well as from the ventral aspect of the tibia in sauropsids, although the femoral origin of the medial head is apomorphic for lepidosaurs (Hutchinson, 2002). The area of insertion is different between the avian and non‐avian muscles, probably caused by the loss of MT V in extant avians. These muscles are consistently served by the tibial nerve in lepidosaurs, testudines, and crocodilians. Such conserved patterns suggest these muscles are homologous with one another. In lepidosaurs, there is a rugose, shelf‐like lateral epicondyle on the ventral surface of the lateral femoral condyle, from which m. flexor digitorum longus (femoral head), m. gastrocnemius, and m. peroneus longus arise tendinously (Russell and Bauer, 2008). This origin is generally retained in archosaurs, as seen in the rugose depression lateroproximal to the lateral condyle in extant crocodilians and the tuberculum m. gastrocnemius lateralis in extant avians.

#### 3.2.2 Musculus flexor hallucis longus

Lepidosauria: M. flexor digitorum longus, in part (Russell and Bauer, 2008)
Testudines: M. flexor digitorum longus, in part (Walker, 1973)
Crocodilia: M. flexor digitorum longus (Suzuki *et al*., 2011)
Aves: M. flexor hallucis longus; M. flexor perforatus digiti II; M. flexor perforans et perforatus digiti II; M. flexor perforatus digiti III; M. flexor perforans et perforatus digiti III; M. flexor perforatus digiti IV (Vanden Berge and Zweers, 1993)

*Lepidosauria*: In the dissected *Iguana* and *Varanus*, one belly of m. flexor digitorum longus arising from the lateral epicondyle of the femur distally became fused with the rest of m. flexor digitorum longus, as described by Russell and Bauer (2008). The fused belly distally formed a stout tendon which inserted on the flexor tubercle of each ungual of digits I–IV. This muscle was innervated by the tibial nerve.

*Testudines*: In *Chelydra*, one slip of m. flexor digitorum longus took the origin from the lateroventral aspect of the femur marked by a shallow and broad depression. It fused with the rest of m. flexor digitorum longus distal to the mesotarsal joint to form the flexor plate, from which four insertion tendons extended to the flexor tubercle of each ungual of digits I–IV. This muscle is innervated by the tibial nerve (Walker, 1973).

*Crocodilia*: In *Paleosuchus* and *Crocodylus*, m. flexor digitorum longus arose from the ventral aspect of the lateral condyle of the femur and merged with m. flexor digitorum longus distally to form the flexor plate, as described by the previous studies (Cong *et al*., 1998; Suzuki *et al*., 2011). The flexor plate divided into four stout tendons to insert on the flexor tubercle of each ungual of digits I–IV. This muscle is innervated by the tibial nerve (Ribbing, 1938; Cong *et al*., 1998; Suzuki *et al*., 2011).

*Aves*: In *Gallus* and *Grus*, long digital flexor muscles arose from several facets on the distal end of the fossa poplitea of the femur as well as from the aponeurosis covering the knee joint and inserted on the flexor tubercle of I‐2 and proximoplantar heels of non‐ungual phalanges of digits II–IV. They are innervated by the medial tibial nerve (Vanden Berge, 1975).

*Comparison*: Although the insertion sites of the long digital flexors in avians are different from the muscles of non‐avian sauropsids listed here, the all muscles share the origin on the ventral aspect of the distal femur and are also innervated by the tibial nerve. Based on these characteristics, the muscles listed here are considered homologous with one another.

#### 3.2.3 Musculus flexor digitorum longus

Lepidosauria: M. flexor digitorum longus, in part (Russell and Bauer, 2008)
Testudines: M. flexor digitorum longus, in part (Walker, 1973)
Crocodilia: M. flexor hallucis longus (Suzuki *et al*., 2011)
Aves: M. flexor digitorum longus; M. plantaris (Vanden Berge and Zweers, 1993)

*Lepidosauria*: In *Iguana* and *Varanus*, one head of m. flexor digitorum longus arose from the depression on the medial aspect of the fibula, just distal to the fibular attachment of m. popliteus as described by Russell and Bauer (2008). It distally merged with the remaining part of m. flexor digitorum longus and together inserted on the flexor tubercle of each ungual of digits I–IV. This muscle is innervated by the tibial nerve (Ribbing, 1938).

*Testudines*: In *Chelydra*, the fibular origin of m. flexor digitorum longus was marked by a distinct surface between the lateral margin of the shaft and the longitudinal ridge situated laterally on the ventral aspect. This muscle inserted on the flexor tubercle of each ungual of digits I–IV by the flexor plate. This muscle is innervated by the tibial nerve (Walker, 1973).

*Crocodilia*: In *Paleosuchus* and *Crocodylus*, a large part of m. flexor hallucis longus arose from the flat surface on the ventral aspect of the fibula, with the origin occupying the proximal two thirds of the bone. An additional origin was on the depression on the ventral aspect of the proximal‐most tibial shaft. The insertion was on the flexor tubercles of the unguals of digits I–IV as a part of the flexor plate. This muscle is innervated by the tibial nerve (Gadow, 1882; Cong *et al*., 1998; Suzuki *et al*., 2011).

*Aves*: In *Gallus* and *Grus*, m. flexor digitorum longus arose from the ventral aspects of both the tibia and fibula, the latter of which was marked by a flat surface on the dorsal aspect of the proximal one third and the lateral and ventral aspects of the distal two thirds of the fibular shaft. The insertion tendon of this muscle attached on the flexor tubercles of the unguals of digits II–IV. This muscle acts as a flexor of the ungual phalanges of digits II–IV, but its fusion with m. flexor hallucis longus allows it to contribute to flexion of the ungual of digit I at least in several raptorial species (Goslow, 1972). M. plantaris arose from the depression on the medioplantar aspect of the proximal tibia. It inserted on the hypotarsal sesamoid bone in the sustentaculum (tibial cartilage) as described by Fujioka (1962). These muscles are innervated by the medial tibial nerve (Vanden Berge, 1975).

*Comparison*: The origins on the tibia and fibula, insertions on the unguals, and innervation by the tibial nerve are mostly conserved among sauropsid muscles listed above, suggesting that these are homologous with one another. Hutchinson (2002) regarded six more muscles arising from the distal end of the femur as homologues of non‐avian m. flexor digitorum longus, although the latter muscle arises from the ventral aspects of the tibia and fibula. These muscles arising from the femur are regarded as homologues of non‐avian m. flexor hallucis longus in the present study (see above).

#### 3.2.4 Musculus pronator profundus

Lepidosauria: M. pronator profundus (Russell and Bauer, 2008)
Testudines: M. pronator profundus (Walker, 1973)
Crocodilia: M. pronator profundus (Suzuki *et al*., 2011)

*Lepidosauria*: In *Iguana* and *Varanus*, m. pronator profundus arose from the distal two thirds of the ventral and medial aspects of the fibular shaft as described by Russell and Bauer (2008). The origin was marked by a flat surface on the ventral aspect of the fibula. This muscle inserted on the rugose surfaces or swellings on the proximomedial margins of the plantar aspects of MTs I–III. In *Varanus*, this muscle was innervated by the tibial nerve and the plantar nerve.

*Testudines*: In *Chelydra*, m. pronator profundus arose from the entire length of the ventral surface of the fibula and the adjacent portion of the astragalocalcaneum and inserted on the proximolateral margin of the plantar aspect of MT I as well as on the adjacent area on DTs I–II, as described on *Trachemys* by Walker (1973). The medial and lateral margins of the fibular origin were marked by longitudinal ridges on the ventral aspect of the shaft. It is innervated by the tibial nerve (Walker, 1973).

*Crocodilia*: In *Paleosuchus* and *Crocodylus*, m. pronator profundus had two origins, one on the lateral aspect of the tibia and the other on the medial aspect of the fibula, occupying the almost entire length of each bone, as described by Suzuki *et al*. (2011). The area of origin on the tibia was a flat surface between longitudinal stout ridges on the dorsolateral and lateral margins on the ventral aspect of the shaft. This muscle inserted on a narrow and rugose swelling on the proximomedial margin of the plantar aspect of MT I. This muscle is innervated by the tibial nerve (Gadow, 1882; Cong *et al*., 1998; Suzuki *et al*., 2011).

*Aves*: This muscle is apparently absent in avians.

*Comparison*: The origin on the ventral aspect of the fibula and insertion on the plantar aspect of MT I are conserved among non‐avian muscles listed above, suggesting that these are homologous with one another. The innervation pattern is also similar to each other except for the additional innervation by the plantar nerve in lepidosaurs. The origin is situated medially to that of m. flexor digitorum longus in testudines and crocodilians, whereas the relative positions of these origins are reversed in lepidosaurs. Despite such difference, the homology of these muscles accepted in the previous study (Hutchinson, 2002) is still the most plausible hypothesis and is thus adopted in the present study.

#### 3.2.5 Musculus fibulocalcaneous

Lepidosauria: M. abductor digiti quinti (Russell and Bauer, 2008)
Crocodilia: M. fibulocalcaneous (Suzuki *et al*., 2011)

*Lepidosauria*: In *Iguana* and *Varanus*, m. abductor digiti quinti arose from the distal margin of the plantar surface of the calcaneal tuber and inserted on the plantar surface of MT V between the medial and lateral tubercles, as described by Russell and Bauer (2008). The area of origin was marked by a smooth surface in contrast to the rugose surface covering the remaining area of the plantar surface of the calcaneal tuber. This muscle is innervated by the planter nerve (Ribbing, 1938).

*Testudines*: This muscle is apparently absent in testudines.

*Crocodilia*: In *Paleosuchus* and *Crocodylus*, m. fibulocalcaneus arose from the ventral aspect of the distal one third of the fibula and inserted on the dorsal surface of the calcaneal tuber, as described in previous studies (Brinkman, 1980; Cong *et al*., 1998; Suzuki *et al*., 2011). The area of origin was marked by a flat surface. This muscle is innervated by the tibial nerve in *Crocodylus* (Ribbing, 1938) and *Alligator* (Gadow, 1882; Cong *et al*., 1998) and the planter nerve in *Caiman* (Suzuki *et al*., 2011).

*Aves*: This muscle is apparently absent in avians.

*Comparison*: M. fibulocalcaneous in crocodilians has previously been described as a homologue of m. abductor digiti quinti of lepidosaurs (Ribbing, 1938) probably because of the similarity in their morphology. Both of these muscles occupy the lateral margin of the deepest layer of the ankle musculature and insert on each distally adjacent bone. The nerve supplying from the distal side of m. fibulocalcaneous suggests that the muscle was originally situated in a more distal portion of the pes in crocodilians (Suzuki *et al*., 2011).

#### 3.2.6 Musculus flexor digitorum brevis superficialis

Lepidosauria: Mm. flexores digitores breves; the calcaneal portion of the plantar component of m. flexor digitorum longus (pfdl. calc.; Russell and Bauer, 2008)
Testudines: Plantar aponeurosis formed by m. gastrocnemius (Walker, 1973)
Crocodilia: M. flexor digitorum brevis superficialis (Suzuki *et al*., 2011)

*Lepidosauria*: In *Iguana* and *Varanus*, mm. flexor digitores breves arose from the tendon of m. femoral gastrocnemius (deep layer of the plantar aponeurosis) as well as from MT V and inserted on the non‐ungual phalanges of digits I–III, as described by Russell and Bauer (2008). The calcaneal portion of the plantar component of the m. flexor digitorum longus (pfdl. calc.) arose from the medial aspect of the calcaneal tuber and inserted plantarly on non‐ungual phalanges of digit IV. These muscles are innervated by the tibial nerve in *Tupinambis* (Ribbing, 1938) and *Varanus* (present observation) and by the plantar nerve in *Hydrosaurus* (Gadow, 1882). Some parts of the plantar aponeurosis unite with tendons of this muscle (Russell, 1993).

*Testudines*: In *Chelydra*, there was a plantar aponeurosis formed by the distal portion of m. gastrocnemius, which inserted on the medial margin of digit I and the proximoplantar heels of II‐1, III‐1, and IV‐1 (Walker, 1973).

*Crocodilia*: In *Paleosuchus* and *Crocodylus*, m. flexor digitorum brevis superficialis consisted of two bellies. The main belly of this muscle arose from the insertion tendon of m. gastrocnemius (plantar aponeurosis), MT V, and astragalus, and inserted on the bases of the non‐ungual phalanges of digits I–III, as described by previous studies (Cong *et al*., 1998; Suzuki *et al*., 2011). According to Suzuki *et al*. (2011), most of the plantar aponeurosis unites with this muscle. The second belly inserting on digit IV, called m. flexor digiti quarti brevis in Cong *et al*. (1998), was separate from the main belly, arising from the distolateral margin of the calcaneum and passed through the sulcus between the medial and lateral plantar tubera of MT V. This muscle is innervated by the tibial nerve in *Alligator* (Gadow, 1882) and by the plantar nerve in *Caiman* (Suzuki *et al*., 2011). The origin on MT V was marked by a longitudinal ridge or bulge on the bone.

*Aves*: This muscle is apparently absent in avians.

*Comparison*: The main and second bellies of the crocodilian m. flexor digitorum brevis superficialis appear to be homologues of the lepidosaurian m. flexores digitores breves and pfdl. calc., respectively. The main belly commonly arises from the insertion tendon of m. gastrocnemius, namely, the plantar aponeurosis, as well as from MT V and inserts on digits I–III. The second belly commonly passes through the plantar aspect of MT V and inserts on digit IV. Furthermore, the innervation pattern similarly varies between the tibial nerve and the plantar nerve. Although there are no fleshy fibers, the plantar aponeurosis observed in testudines that possesses a very similar pattern of the origin and insertion as those muscles in lepidosaurians and crocodilians suggests the homologous relationship between these tissues.

#### 3.2.7 Musculus flexor digitorum brevis profundus

Lepidosauria: fifth metatarsal portion of the plantar component of m. flexor digitorum longus (pfdl. met. 5; Russell and Bauer, 2008)
Crocodilia: M. flexor digitorum brevis profundus (Suzuki *et al*., 2011)

*Lepidosauria*: In the examined *Iguana* and *Varanus*, the MT V portion of the plantar component of the m. flexor digitorum longus (pfdl. met. 5) arose from the concavity on the medial aspect of MT V. It merged with m. flexor digitorum longus tendon inserting on digits II–IV as described by Russell and Bauer (2008). This muscle is innervated by both the tibial and plantar nerves in *Hydrosaurus* (Gadow, 1882) and *Varanus* (present observation) or only by the tibial nerve in *Tupinambis* (Ribbing, 1938).

*Testudines*: This muscle is apparently absent in testudines.

*Crocodilia*: In the examined *Paleosuchus* and *Crocodylus*, m. flexor digitorum brevis profundus arose from the medial and lateral margins of the calcaneal tuber and the concavity on the distomedial margin of the plantar aspect of MT V. This muscle forms the flexor plate with the tendons of m. flexor hallucis longus and m. flexor digitorum longus (Cong *et al*., 1998; Suzuki *et al*., 2011). Distally the flexor plate was divided into three tendons and inserted on the flexor tubercles of the unguals of digits I–III. In crocodilians, two innervation patterns are reported, either only by the plantar nerve (Cong *et al*., 1998; Suzuki *et al*., 2011) or by both the plantar and tibial nerves (Gadow, 1882; Ribbing, 1938).

*Aves*: This muscle is apparently absent in avians.

*Comparison*: M. flexor digitorum brevis profundus in crocodilians has previously been described as a homologue of pfdl. met. 5 of lepidosaurs (Gadow, 1882; Ribbing, 1938). This homology is adopted in the present study because both of these muscles share the origin on MT V and insertion on the unguals of three digits. Because of apparent plasticity of the innervation pattern both in crocodilians and lepidosaurs, the homology inferred here is solely based on the origin/insertion patterns of the muscles.

#### 3.2.8 Musculi lumbricales

Lepidosauria: Mm. lumbricales (Russell and Bauer, 2008)
Testudines: M. flexor digitorum communis sublimis (Walker, 1973)
Crocodilia: Mm. lumbricales (Suzuki *et al*., 2011)

*Lepidosauria*: In *Iguana* and *Varanus*, mm. lumbricales consisted of two bellies: the medial belly arose from the aponeurosis between the insertion tendons of m. flexor digitorum longus inserting on digits II–III, whereas the lateral belly arose from the lateral margin of the insertion tendon of m. flexor digitorum longus inserting on digit III, as described by Russell and Bauer (2008). The medial belly inserted on the proximoplantar heel of each non‐ungual phalanx of digit III. On the other hand, the lateral belly inserted on non‐ungual phalanges of digit IV in the same manner. The plantar nerve innervated the medial belly in *Varanus*, whereas the lateral belly was innervated by the tibial nerve.

*Testudines*: In *Chelydra*, m. flexor digitorum communis sublimis arose from the plantar surface of the flexor plate of m. flexor digitorum longus and inserted on the medial side of the flexor aponeurosis of digits I–IV as described by Walker (1973). This muscle is innervated by the tibial nerve (Walker, 1973).

*Crocodilia*: In *Paleosuchus* and *Crocodylus*, mm. lumbricales arose from the flexor plate consisting of m. flexor digitorum longus and m. flexor hallucis longus and inserted on the lateral aspects of the proximal‐most phalanges of digits I–IV, as described by Suzuki *et al*. (2011). In both taxa, the origin of this muscle was situated on the dorsal side of the flexor plate. The plantar nerve innervates this muscle (Gadow, 1882; Cong *et al*., 1998; Suzuki *et al*., 2011).

*Aves*: This muscle is apparently absent in avians.

*Comparison*: Mm. lumbricales of both lepidosaurs and crocodilians and m. flexor digitorum communis sublimis of testudines are most likely homologous with each other based on the similar areas of origin. Whereas the topological relationship with the flexor plate reverses between testudines and crocodilians, the intermediate condition between them seen in lepidosaurs might represent the ancestral condition for Sauropsida.

#### 3.2.9 Musculus lumbricalis profundus

Lepidosauria: Aponeurosis of the plantar component of the m. flexor digitorum longus (pfdl. ap.; Russell and Bauer, 2008)
Testudines: Mm. lumbricales (Walker, 1973)
Crocodilia: M. lumbricalis profundus (Suzuki *et al*., 2011)
Aves: M. lumbricalis (Vanden Berge and Zweers, 1993)

*Lepidosauria*: In lepidosaurs including *Sphenodon*, the plantar component of the m. flexor digitorum longus (pfdl. ap.) arises from the dorsal aspects of the insertion tendons of m. flexor digitorum longus and inserts on the proximoplantar heels of II‐1, III‐1 and IV‐1 (Russell and Bauer, 2008).

*Testudines*: In the dissected *Chelydra*, mm. lumbricales arose from the dorsal surface of the flexor plate and divided into three slips. Two of them inserted on the medioplantar margins of non‐ungual phalanges of digits IV and V. Another slip inserted on both medioplantar and lateroplantar margins of the penultimate phalanx of digit II. Although the insertion sites of this muscle are slightly different from those of *Trachemys*, the area of origin is very similar with each other. This muscle is innervated by the tibial nerve (Walker, 1973).

*Crocodilia*: M. lumbricalis profundus has been described as arising from the ligament between the calcaneum and MT V (*Ligamentum plantare longum* in Cong *et al*., 1998) and being distally united with the insertion tendon of the lumbricales inserting on digits I and II (Suzuki *et al*., 2011). This muscle is innervated by the plantar nerve (Suzuki *et al*., 2011).

*Aves*: M. lumbricalis arises from the flexor plate consisting of the long digital flexors homologous to the crocodilian m. flexor digitorum longus and m. flexor hallucis longus (see above) and inserts plantarly on III‐1 and IV‐1 (Vanden Berge, 1975; Gangl *et al*., 2004). In *Apteryx*, this muscle distally attaches to the proximoplantar heels of II‐1, III‐1, and IV‐1 (McGowan, 1979). In *Eudromia*, this muscle inserts on both medial and lateral sides of the proximal‐most phalanges of each digit (Suzuki *et al*., 2014). This muscle is weakly developed in gallinaceous avians, represented by an indistinct bundle of fleshy fibers lying deep to the long digital flexors (Vanden Berge, 1975). In *Columba*, this muscle inserts only on digits III and IV (Cracraft, 1971). This muscle is innervated by the lateral plantar nerve (Vanden Berge, 1975).

*Comparison*: The insertion sites of these muscles vary among these clades to a certain extent, but nonetheless are commonly situated plantarly on non‐ungual phalanges. The origins of pfdl. ap. in lepidosaurs and mm. lumbricales in testudines are on the flexor plate. Although the origin of m. lumbricalis profundus is situated on the ligament in crocodilians, it is plantarly adjacent to the flexor plate as are those muscles in other sauropsids. These muscles are consistently innervated by the tibial nerve or its branch, the plantar nerve.

#### 3.2.10 Musculus flexor hallucis brevis

Lepidosauria: M. flexor hallucis (Russell and Bauer, 2008)
Testudines: Mm. interossei plantares, in part (Walker, 1973)
Crocodilia: M. flexor hallucis brevis superficialis; M. flexor hallucis brevis profundus (Suzuki *et al*., 2011)

*Lepidosauria*: In the examined *Iguana* and *Varanus*, m. flexor hallucis arose tendinously from the plantar tubercle of DT IV and inserted plantarly on the proximoplantar heel of I‐1. This muscle is innervated by the tibial nerve in *Varanus* (present observation) and *Tupinambis* (Ribbing, 1938).

*Testudines*: In *Chelydra*, a portion of mm. interossei plantares arose from the depression on the plantar aspect of DT I and inserted on the proximoplantar heel of I‐1. This muscle is innervated by the tibial nerve (Walker, 1973).

*Crocodilia*: In *Paleosuchus* and *Crocodylus*, m. flexor hallucis brevis superficialis and profundus arose from the ligamentum plantare longum and inserted on the longitudinal depression on the plantar surface of MT I and the proximoplantar heel of I‐1. These muscles are innervated by the plantar nerve (Gadow, 1882; Cong *et al*., 1998; Suzuki *et al*., 2011).

*Aves*: This muscle is apparently absent in avians.

*Comparison*: M. flexor hallucis in Lepidosauria is most likely to be homologous with m. flexor hallucis brevis in Crocodilia based on the shared position of the insertion as well as similar positions of the origins. Ribbing (1938) regarded this muscle as a portion of the muscle (mm. contrahentes) homologous between lepidosaurs and crocodilians, although this homology is not supported by the innervation pattern.

#### 3.2.11 Musculus adductor digiti V

*Lepidosauria*: M. adductor digiti quinti (Russell and Bauer, 2008)

*Lepidosauria*: In *Iguana* and *Varanus*, m. adductor digiti quinti arose from the lateral aspect of the medial plantar tubercle of MT V and inserts on each phalanx of digit V except for the ungual, as described by Russell and Bauer (2008). The innervation pattern of this muscle has not been described.

*Testudines*,*Crocodilia*,*and Aves*: This muscle is apparently absent in testudines, crocodilians, and avians.

*Comparison*: This muscle is absent in crocodilians and avians because of the absence of digit V. In testudines, although digit V is present, no homologue of this muscle is recognized. It is still unknown whether the presence of this muscle is the ancestral condition for Sauropsida or a lepidosaurian autapomorphy.

#### 3.2.12 Musculi contrahentes

Lepidosauria: Mm. contrahentes (Russell and Bauer, 2008)
Testudines: Mm. interossei plantares, in part (Walker, 1973)
Crocodilia: M. flexor digiti II; M. flexor digiti III; M. flexor digiti IV (Suzuki *et al*., 2011)

*Lepidosauria*: In the examined *Iguana* and *Varanus*, slips of mm. contrahentes inserting on digits I–III had a common origin on a small tubercle on the medial margin of the shaft of MT V (contra “proximomedial end of the shaft” in Russell and Bauer, 2008). Another distinct slip inserting on digit IV arose from the proximal aspect of the medioplantar margin of MT IV. Each slip of mm. contrahentes has been described as inserting on the lateral aspect of the proximal shaft of the first phalanx of its respective digit (Russell and Bauer, 2008).

*Testudines*: In *Chelydra*, a portion of mm. interossei plantares arose from the distoplantar margin of MT V and inserted on lateroproximal margins of the proximal phalanges of digits I–IV. This portion is innervated by the tibial nerve (Walker, 1973).

*Crocodilia*: In the dissected *Paleosuchus* and *Crocodylus*, the proximal‐most portions of bellies associated with the second (m. flexor digiti II) and third digits (m. flexor digiti III), as well as the proximomedial part of the slip associated with the fourth digit (m. flexor digiti IV), proximally shared the common origin on the plantar aspects of DTs III and IV. The area of origin was marked by a depression in *Paleosuchus*, whereas such depression was not observed in *Crocodylus* probably because of the immaturity of the examined specimen. M. flexor digiti IV has an additional origin on the distal end of MT V (Cong *et al*., 1998; Suzuki *et al*., 2011). These muscles became separated from each other immediately distal to the origin and inserted on the medioplantar surfaces of their respective MTs.

*Aves*: This muscle is apparently absent in avians.

*Comparison*: These muscles are commonly situated in the deepest layer of the plantar mass, except for the muscles homologous to the crocodilian mm. interossei plantares. The origins of these muscles are commonly situated on DT IV in Testudines and Crocodilia, whereas the origins on MT V are shared between Lepidosauria and Crocodilia. The insertion mode on the lateral aspect of the proximal phalanges of digits I–IV is mostly similar between lepidosaurs and testudines, whereas the insertion is on MTs II–IV in crocodilians. Although these attachment patterns are highly variable, these muscles are here regarded as homologous with each other based on the facts that these muscles comprise the deepest layer of the planter muscles and that they share the innervation by the tibial nerve or its branch, the plantar nerve.

#### 3.2.13 Musculus adductor hallucis plantaris

Lepidosauria: Mm. interossei plantares, in part (Russell and Bauer, 2008)
Testudines: Mm. interossei plantares, in part (Walker, 1973)
Crocodilia: M. adductor hallucis plantaris (Suzuki *et al*., 2011)
Aves: M. flexor hallucis brevis (Vanden Berge and Zweers, 1993)

*Lepidosauria*: In *Iguana* and *Varanus*, a portion of mm. interossei plantares arose from the dorsomedial surface of the proximal MT II and inserted laterally on the distal shaft of MT I and proximoplantar heel of I‐1 (contra ventromesial aspect of MT I as described by Russell and Bauer, 2008). The innervation pattern has not been described for this muscle.

*Testudines*: In *Chelydra*, a superficial slip and a deep slip of mm. interossei plantares arose from the depression on the medial aspect of the proximal portion of MT II, and inserted on the lateral aspect of I‐1 and MT I, respectively. This muscle is innervated by the tibial nerve (Walker, 1973).

*Crocodilia*: In *Paleosuchus* and *Crocodylus*, m. adductor hallucis plantaris arose from the depression on the mediodorsal aspect of MT II near its proximal end and inserted on the broad, shallow plantar depression of MT I. This muscle is innervated by the planter nerve (Suzuki *et al*., 2011).

*Aves*: In *Gallus* and *Grus*, m. flexor hallucis brevis arose from the medial part of the sulcus flexorius, and inserted on the proximoplantar heel of I‐1. This muscle is innervated by the lateral plantar nerve (Vanden Berge, 1975).

*Comparison*: The muscles in non‐avian sauropsids listed above share the origin on the medial aspect of MT II. This is topologically similar to the area of origin of the avian m. flexor hallucis brevis. Although the insertions vary among taxa (MT I and/or I‐1), all of these muscles are innervated by the tibial nerve or its branch and their mechanical functions are also similar to each other. Based on these characteristics, the muscles listed here are considered homologous with one another.

#### 3.2.14 Musculus interosseous plantaris digiti II

Lepidosauria: Mm. interossei dorsales, in part; Mm. interossei plantares, in part (Russell and Bauer, 2008)
Testudines: Mm. interossei plantares, in part (Walker, 1973)
Crocodilia: M. interosseous plantaris digiti II (Suzuki *et al*., 2011)
Aves: M. adductor digiti II (Vanden Berge and Zweers, 1993)

*Lepidosauria*: In *Iguana* and *Varanus*, a muscle slip consisting of parts of mm. interossei dorsales and mm. interossei plantares took the origin on the dorsomedial and medioplantar (contra ‘ventrolateral’ for mm. interossei plantares in Russell and Bauer, 2008) surfaces of the proximal shaft of MT III and inserted on the lateroplantar surface (contra ‘ventromesial’ for mm. interossei plantares and ‘mesial’ for m. interossei dorsales in Russell and Bauer, 2008) of the almost entire shaft of MT II. The innervation pattern of this muscle has not been described in lepidosaurs.

*Testudines*: In *Chelydra*, a portion of mm. interossei plantares arose from the depression on the medial aspect of MT III and inserted laterally on MT II. This muscle is innervated by the tibial nerve (Walker, 1973).

*Crocodilia*: In *Paleosuchus* and *Crocodylus*, the origin of m. interosseous plantaris digiti II was similar to that of mm. interossei dorsales in lepidosaurs and is characterized by a shallow depression, and inserted fleshly on the plantar aspect between the plantar ridge and lateral margin of MT II and tendinously on the lateroplantar margin near its distal end. This muscle is innervated by the plantar nerve (Gadow, 1882; Cong *et al*., 1998; Suzuki *et al*., 2011).

*Aves*: In *Gallus* and *Grus*, m. adductor digiti II arose from the central part of the distal sulcus flexorius and inserted on a small tubercle on the lateroplantar margin of the proximal end of II‐1. This muscle is innervated by the lateral plantar nerve (Vanden Berge, 1975).

*Comparison*: The origins of these muscles are commonly on the medial aspect of MT III among non‐avian sauropsids. The insertion pattern exhibits apparent plasticity. The insertion on MT II is shared only between Lepidosauria and Crocodilia, whereas that on digit II is common in sauropsids except for Crocodilia. The innervation pattern is conserved among the muscles listed here and they are thus considered homologous with one another.

#### 3.2.15 Musculus interosseous plantaris digiti III

Lepidosauria: Mm. interossei dorsales, in part; Mm. interossei plantares, in part (Russell and Bauer, 2008)
Testudines: Mm. interossei plantares, in part (Walker, 1973)
Crocodilia: M. interosseous plantaris digiti III (Suzuki *et al*., 2011)

*Lepidosauria*: In *Iguana* and *Varanus*, a slip consisting of parts of mm. interossei dorsales and plantares took origins on the medial and plantar (contra ‘ventrolateral’ for mm. interossei plantares in Russell and Bauer, 2008) surfaces of the proximal two thirds of MT IV and inserted on the lateroplantar surface (contra ‘ventromesial’ for mm. interossei plantares in Russell and Bauer, 2008) of the almost entire shaft of MT III. The innervation pattern has not been described for this muscle in lepidosaurs.

*Testudines*: In *Chelydra*, a portion of mm. interossei plantares arose from the depression on the medial aspect of MT IV and inserted laterally on MT III. The same muscle has an additional origin on DT IV and inserts on III‐1 in *Trachemys* (Walker, 1973). This muscle is innervated by the tibial nerve (Walker, 1973).

*Crocodilia*: In *Paleosuchus*, m. interosseous plantaris digiti III arose from the depressions on the dorsomedial and medioplantar aspects of proximal MT IV and inserted on the lateroplantar surface of MT III by a tendon as well as on the lateroplantar margin of the proximal end of III‐1. The tendinous insertion on MT III left a distinct depression on the distal end of the attachment area on the bone. The insertion on MT III is absent in *Crocodylus* and *Caiman* (Suzuki *et al*., 2011). Correspondingly, a flat surface lateral to the plantar ridge was found absent in the specimen of *Crocodylus* dissected in the present study.

*Aves*: This muscle is apparently absent in avians.

*Comparison*: These muscles listed here commonly arise from the proximomedial portion of MT IV and insert on MT III in lepidosaurs and crocodilians. Although the insertion on MT III is absent in Testudines, the one on III‐1 is shared between this clade and Crocodilia. All of these muscles are commonly innervated by the tibial nerve or plantar nerve branching from it.

#### 3.2.16 Musculus abductor digiti IV

Lepidosauria: Mm. extensores digitores breves, in part (Russell and Bauer, 2008)
Testudines: Mm. interossei plantares, in part (Walker, 1973)
Crocodilia: M. abductor digiti IV dorsalis; M. abductor digiti IV plantaris (Suzuki *et al*., 2011)
Aves: M. abductor digiti IV (Vanden Berge and Zweers, 1993)

*Lepidosauria*: In *Iguana*, the abductors of digit IV were represented by two parts of mm. extensores digitorum breves. Fleshy origins of one part were on the dorsolateral aspect of the distal fibula, just lateral to the origin of m. adductor et extensor hallucis indicus, and the dorsomedial surface of MT V (distal to the proximal articular surface for DT IV, and dorsal to the origin of m. flexor digitorum brevis superficialis). The other part took its origin on the laterodorsal margin of the proximal end of MT IV by a small accessory tendon. These two parts merged immediately. Distally, two insertion tendons emerged: one inserted on the proximal ends of V‐2, 3 and 4, whereas the other on the proximolateral margin of each phalanx of digit IV. In addition, a tendon that arose from the insertion of m. femorotibial gastrocnemius on MT V and extended through the ventral part of the joint capsule of the metatarsophalangeal joint of the digit V was united with the above tendon inserting on digit IV.

*Testudines*: In *Chelydra*, a portion of mm. interossei plantares arose from the depression on the distal aspect of the medial portion of MT V and inserted laterally on the proximal shaft of IV‐1. This muscle is innervated by the tibial nerve (Walker, 1973).

*Crocodilia*: In *Crocodylus* and *Paleosuchus*, m. abductor digiti IV dorsalis and m. abductor digiti IV plantaris originated from the mediodistal margin of the dorsal aspect and the distal end of MT V. These muscles merged with the medial aspect of the dorsal aponeurosis of digit IV and inserted on the proximolateral margins of phalanges of digit IV and on a flat surface between the plantar ridge and the medial margin of MT IV. An accessory origin is known in *Alligator* (dorsal aspect of DT IV; Cong *et al*., 1998) and *Caiman* (calcaneal tuber; Suzuki *et al*., 2011). This muscle is innervated by the plantar nerve (Suzuki *et al*., 2011).

*Aves*: In *Gallus* and *Grus*, m. abductor digiti IV arose from the lateral portion of the sulcus flexorius and inserted on the lateroproximal margin of IV‐1. The insertion was marked as a tubercle projecting plantarly. This muscle conducts abduction as well as extension of this digit to a slight extent, and is innervated by the lateral plantar nerve (Vanden Berge, 1975).

*Comparison*: The origin on MT V, insertion on digit IV, and innervation by the tibial nerve or plantar nerve branching from it are all conserved among non‐avian sauropsids, suggesting the homology among the muscles listed here. In avians, because there is no muscle inserting laterally on digit IV other than m. adductor digiti IV, the latter muscle is most likely homologous to the non‐avian muscles listed here.

## 4 DISCUSSION

### 4.1 Homology of m. extensor digitorum longus and m. tibialis cranialis

**Figure 10:**
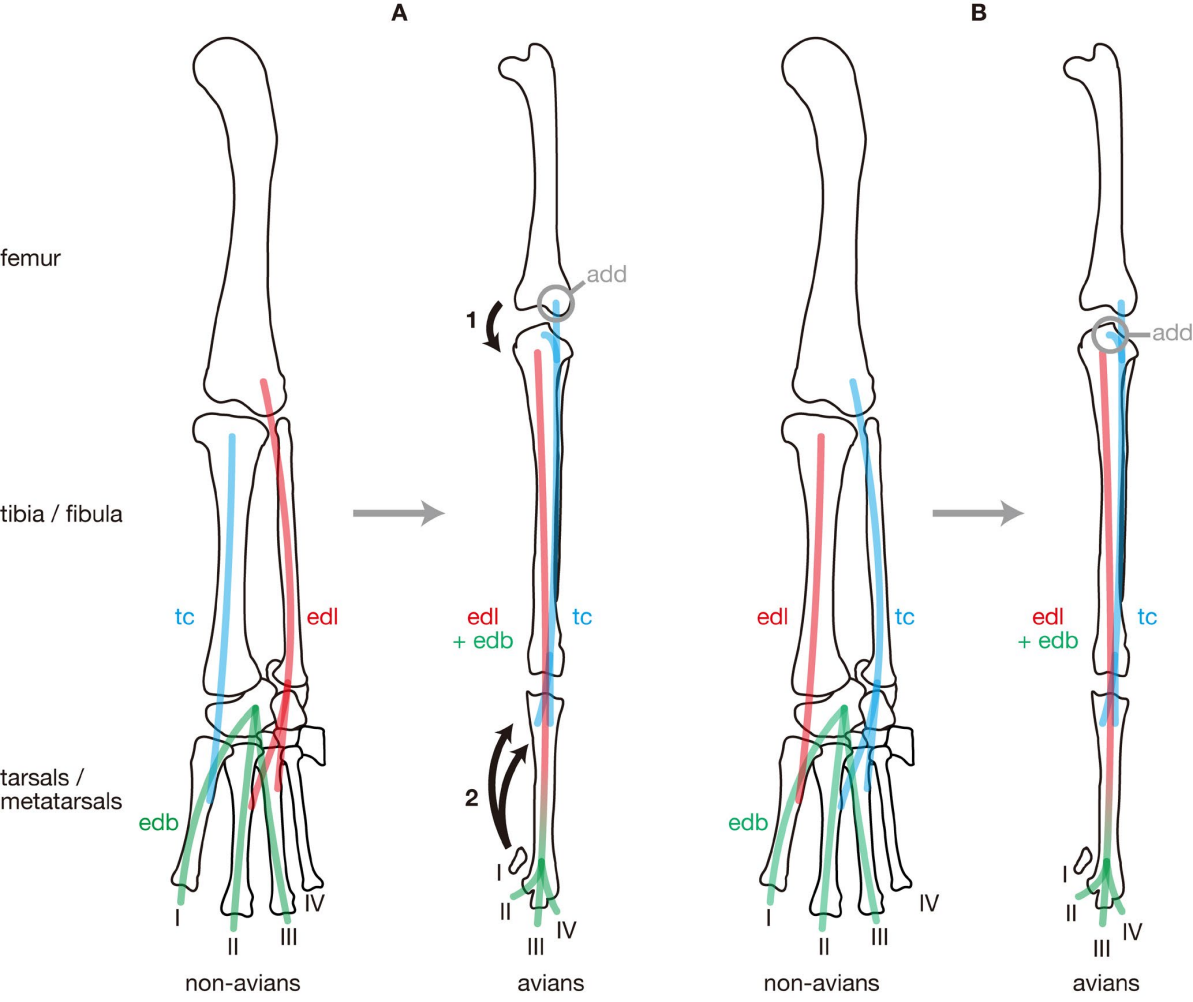
Schematic illustrations comparing hypotheses on the homology of the avian m. tibialis cranialis. (a) Traditional hypothesis accepted in previous studies (e.g. Carrano and Hutchinson, 2002; Hutchinson, 2002). (b) New hypothesis proposed in the present study. Changes in the muscle attachment required for the traditional hypothesis are indicated by numbers in (a). 1, Displacement of the origin of m. extensor digitorum longus across the knee joint; 2, Loss of the insertion of m. tibialis cranialis from MT I. Note that these changes are not necessary for the new hypothesis as shown in (b). Abbreviations: add, addition of the origin of m. tibialis cranialis; edl, m. extensor digitorum longus; edb, m. extensor digitorum brevis; tc, m. tibialis cranialis; I–IV, digits I–IV

The homology between the non‐avian ‘m. extensor digitorum longus’ and the avian m. tibialis cranialis, as well as the non‐avian ‘m. tibialis anterior’ and the avian m. extensor digitorum longus, is proposed in the present study (Figure 10; Table 2). This hypothesis contradicts a traditional hypothesis that ‘m. tibialis anterior’ of non‐avian sauropsids is a homologue of the avian m. tibialis cranialis accepted by many previous studies (Dilkes, 2000; Hutchinson, 2002; Schachner *et al*., 2011). However, Dilkes (2000) expressed some doubt on the traditional hypothesis, and the possibility of an alternative homology that is the one proposed in this study was suggested by Hutchinson (2002). These homology hypotheses are discussed further in this section.

The non‐avian ‘m. tibialis anterior’ arises from the dorsomedial aspect of the tibial shaft and inserts mainly on MT I, whereas the avian m. tibialis cranialis arises from the lateral condyle of the femur (fovea tendinis m. tibialis cranialis) and the proximal extremity of the cnemial crest of the tibia and inserts on the dorsal aspect of the tarsometatarsus. Although both muscles are commonly innervated by the fibular nerve, the positions of both origin and insertion are clearly different between these non‐avian and avian muscles. Such difference is also present between the non‐avian ‘m. extensor digitorum longus’ in traditional terminology and the avian m. extensor digitorum longus. The non‐avian ‘m. extensor digitorum longus’ arises from the laterodorsal aspect of the distal femur and inserts on MTs II–IV, whereas the avian m. extensor digitorum longus arises from the broad dorsolateral surface of the tibia (sulcus intercnemialis) and inserts on the extensor tubercle (tuberculum extensorium) of each ungual of digits II–IV in avians. In order to explain the difference in the insertion site between these two, putatively homologous muscles, Carrano and Hutchinson (2002) proposed a hypothesis that the avian m. extensor digitorum longus has been formed by the fusion of the non‐avian ‘m. extensor digitorum longus’ and m. extensor digitorum brevis because the latter muscle is absent as a distinct muscle in extant avians. If this hypothesis is accepted, however, the ankle flexors would need to be homologous with each other despite the difference in the site of origin. In addition, although the non‐avian ‘m. tibialis anterior’ is situated medially to ‘m extensor digitorum longus’ (Cong *et al*., 1998, fig. 118), the avian m. tibialis cranialis is situated laterally to m. extensor digitorum longus (Fujioka, 1962, fig. 4b).

Hutchinson (2002) regarded the homology hypothesis between the avian m. tibialis cranialis and non‐avian ‘m. extensor digitorum longus’ “slightly more parsimonious” than the one homologizing the former muscle with the non‐avian ‘m. tibialis anterior.’ The former hypothesis is favored in the present study because it requires only one evolutionary change in their origins, whereas the traditional homology hypothesis would force three changes in their origins (Figure 10). A conspicuous change in the insertion of m. extensor digitorum longus required for the new hypothesis is resolved by postulating the fusion of this muscle with m. extensor digitorum brevis (Figure 10B) as is the case with non‐avian ‘m. extensor digitorum longus’ in the traditional hypothesis.

### 4.2 Interosseous muscles and short digital extensor/flexors

In lepidosaurs and testudines, the dorsal interosseous muscles (mm. interossei dorsales digiti II–IV) have been regarded as a part of the complex of short digital extensors (Walker, 1973; Russell and Bauer, 2008). However, as a result of the dissections conducted for the present study, clearly distinct muscle slips that arise from the proximolateral aspect of each metatarsal medially adjacent to the digit of insertion and accompany stout tendons were commonly observed in non‐avians. These slips became distally fused with the short extensors to a certain degree. However, because each of these slips comprises the medial portion of each digital extensor, most of their fibers insert on the proximomedial margins of the phalanges and likely serve as abductors for digits II–III and an adductor for digit IV as well if they act independently. Such distinction of slips within the deep dorsal musculature, mm. interossei dorsales digiti II–IV, were previously unknown in non‐archosaurian sauropsids but facilitates comparison of the short dorsal musculature between non‐avian sauropsids and avians, thus, contributing to a much better understanding of the phylogenetic origin of the avian short digital extensors.

Similarly, the plantar interosseous muscles (mm. interossei plantares digiti II et III and m. abductor digiti IV) were previously regarded as portions of the complex of short digital flexors in testudines (Walker, 1973). Although clearly distinct slips arise from the proximomedial aspect of each metatarsal laterally adjacent to the digit of insertion, these slips merge with larger slips of mm. contrahentes arising from the distal tarsal and metatarsal of each digit of insertion. The same or similar names have been applied to these non‐homologous muscles, such as mm. interossei dorsales in lepidosaurs and mm. interossei plantares in lepidosaurs and testudines (Walker, 1973; Russell and Bauer, 2008) probably due to the small size of the slips of the interosseous muscles relative to mm. contrahentes with which they merge and the complexity of the plantar musculature, as well as to the fact that very few comparative studies have been conducted between testudines and other sauropsids.

### 4.3 Comparison of pedal musculatures between non‐avian sauropsids and avians

Although hypotheses of homology were proposed for all pedal muscles among non‐avian sauropsids in the present study, avians were considered as lacking at least 12 of such muscles while possessing 5 additional muscles produced through division of m. flexor digitorum longus. It follows that the plesiomorphic condition of the pedal muscles present in ancestral archosaurs has been retained in the pseudosuchian lineage, whereas many such muscles have been lost and some neomorphic muscles have been acquired in the ornithodiran lineage. After the divergence from the pseudosuchian lineage, ornithodirans acquired a fully erect limb posture and obligate bipedalism as observed in theropod dinosaurs including living avians (Sullivan, 2015). Although the bipedality is also seen in some Triassic pseudosuchians such as poposauroids (Gauthier *et al*., 2011) such forms became extinct at the Triassic–Jurassic boundary, whereas the bipedality has continued to be refined along the ornithodiran line for 230 million years. Such a long evolutionary history of the bipedalism is likely reflected by many apomorphic conditions in the pedal musculature observed in extant avians. Thus, the present homology assessments are the first step toward a comprehensive understanding of the evolution of the morphology and function of the bipedality toward extant Aves. An analysis on the sequence of acquisitions of such apomorphies based on osteological correlates of the pedal muscles in fossil ornithodirans, as has been done on other himdlimb muscles by Hutchinson (2002a, 2002b), would further clarify the evolutionary pattern of the bipedality in this clade.

## 5 CONCLUSIONS

In the present study, the morphology and positions of the origins and insertions of hindlimb muscles attaching to the pedal skeleton of sauropsids including archosaurs were examined and described in detail. Although the detailed morphology of the musculature associated with the pedal skeleton has been described in detail in Gadow (1882), Cong *et al*. (1998), and Suzuki *et al*. (2011) on crocodilians and Fujioka (1962) and Yasuda (2002) on *Gallus gallus*, the details of osteological signatures associated with muscle attachments have not been discussed extensively even for these taxa. In addition, although the pedal musculature has been briefly mentioned in some studies reconstructing the hindlimb musculature in extinct archosaurs (e.g. Dilkes, 2000; Carrano and Hutchinson, 2002; Schachner *et al*., 2011), it has never been discussed or illustrated sufficiently.

The present study provided detailed information on positions of muscle attachments in the pes and morphology of their osteological correlates for the first time throughout major clades of extant sauropsids, leading to detailed homology hypotheses of the pedal muscles among sauropsids. It not only enables comparison of the morphology and functions of the pedal musculature among the major clades of Lepidosauria, Testudines, Crocodilia, and Aves, revealing major changes in this anatomical system having occurred within the ornithodiran lineage, but also serves as the basis for its reconstructions in extinct archosaurian taxa, which have long been neglected in previous studies on their myology and kinematics. Therefore, the information and homology hypotheses presented here serves as important clues for revealing the pattern and process of acquisitions of apomorphic functional characters present in avian hindlimbs such as the erect and digitigrade limb posture and the bipedal locomotion, although these hypotheses still need to be further tested by ontogenetic data and anatomical studies on more taxa.

## AUTHOR CONTRIBUTIONS

Study concept/design and acquisition of data: SH and TT. Data interpretation and drafting of the manuscript: SH. Critical revision of the manuscript and approval of the article: SH and TT.

## Data Availability

The data that support the findings of this study are available from the corresponding author upon reasonable request.
